# Tensorial Principal Component Analysis in Detecting Temporal Trajectories of Purchase Patterns in Loyalty Card Data: Retrospective Cohort Study

**DOI:** 10.2196/44599

**Published:** 2023-12-15

**Authors:** Reija Autio, Joni Virta, Klaus Nordhausen, Mikael Fogelholm, Maijaliisa Erkkola, Jaakko Nevalainen

**Affiliations:** 1 Faculty of Social Sciences (Health Sciences) Tampere University Tampere Finland; 2 Department of Mathematics and Statistics University of Turku Turku Finland; 3 Department of Mathematics and Statistics University of Jyväskylä Jyväskylä Finland; 4 Department of Food and Nutrition University of Helsinki Helsinki Finland

**Keywords:** tensorial data, principal components, loyalty card data, purchase pattern, food expenditure, seasonality, food, diet

## Abstract

**Background:**

Loyalty card data automatically collected by retailers provide an excellent source for evaluating health-related purchase behavior of customers. The data comprise information on every grocery purchase, including expenditures on product groups and the time of purchase for each customer. Such data where customers have an expenditure value for every product group for each time can be formulated as 3D tensorial data.

**Objective:**

This study aimed to use the modern tensorial principal component analysis (PCA) method to uncover the characteristics of health-related purchase patterns from loyalty card data. Another aim was to identify card holders with distinct purchase patterns. We also considered the interpretation, advantages, and challenges of tensorial PCA compared with standard PCA.

**Methods:**

Loyalty card program members from the largest retailer in Finland were invited to participate in this study. Our LoCard data consist of the purchases of 7251 card holders who consented to the use of their data from the year 2016. The purchases were reclassified into 55 product groups and aggregated across 52 weeks. The data were then analyzed using tensorial PCA, allowing us to effectively reduce the time and product group-wise dimensions simultaneously. The augmentation method was used for selecting the suitable number of principal components for the analysis.

**Results:**

Using tensorial PCA, we were able to systematically search for typical food purchasing patterns across time and product groups as well as detect different purchasing behaviors across groups of card holders. For example, we identified customers who purchased large amounts of meat products and separated them further into groups based on time profiles, that is, customers whose purchases of meat remained stable, increased, or decreased throughout the year or varied between seasons of the year.

**Conclusions:**

Using tensorial PCA, we can effectively examine customers’ purchasing behavior in more detail than with traditional methods because it can handle time and product group dimensions simultaneously. When interpreting the results, both time and product dimensions must be considered. In further analyses, these time and product groups can be directly associated with additional consumer characteristics such as socioeconomic and demographic predictors of dietary patterns. In addition, they can be linked to external factors that impact grocery purchases such as inflation and unexpected pandemics. This enables us to identify what types of people have specific purchasing patterns, which can help in the development of ways in which consumers can be steered toward making healthier food choices.

## Introduction

Loyalty card data comprise a big data source [1], which is becoming increasingly important in research [2-5]. Loyalty cards are electronic customer cards used in grocery retailing that automatically register grocery expenditure per purchased item every time the customer swipes their card at the store. Card holder–speciﬁc loyalty card data should not be confused with aggregated data such as store-speciﬁc point of sales data; instead, the former provides information on both the product group and the time of each customer’s purchases. The level of details in product groups (eg, amount and price) and time within loyalty card data make it an attractive source of information in, for instance, public health research [2]. Loyalty card data provide new insights into health-related purchase behavior [6-10]. Food, tobacco, and alcohol are well-known risk factors for many chronic diseases; however, their consumption is difficult to measure using traditional methods [11,12]. Using loyalty card data, the purchases of these items can be measured in an automated, detailed, and objective fashion.

Loyalty card data are structured in such a way that individual card holders, expenditures, and the volume of product groups purchased within a single period constitute the usual data matrix. However, when the data are arranged by time of purchase, they are longitudinal, and the third dimension is time, including information on, for example, time trends, seasonality, or change points. Therefore, loyalty card data are longitudinal data, having dimensions in both time and product groups, and can be seen as a tensor.

Tensorial data are multidimensional array data. Vectors are 1D or first-order tensors; matrices are 2D or second-order tensors; and more generally, tensors are generalization of matrices to the n-dimensional space. Tensorial data, images, and videos, for example, are becoming increasingly common. In addition, our loyalty card data resemble a video, which is set up as a dense sequence of images, each detailing the expenditures by product group for all participants during a fixed time. Simultaneously, methods for the analysis of tensor-valued data have developed substantially [13,14].

Although modern measurement systems have increased the dimensionality of data, these dimensions usually include a lot of redundant information within the data. Therefore, in data analysis, often the aim is to decrease the number of dimensions by removing the redundancy without losing the substantial information within the data. Dimension reduction has become a standard tool in exploratory statistics, with the dual goal of reducing the number of variables in a data set and extracting easy-to-interpret latent components for further analysis. Since the introduction of the standard principal component analysis (PCA), a search for uncorrelated subspaces containing a maximal amount of variation [15,16], a vast number of dimension reduction methods for different types of high-dimensional data have been proposed. Dimension reduction for longitudinal data has been studied elsewhere [17] using multivariate functional PCA methodology.

Traditionally, research on the health effects of diet has primarily focused on examining the associations between individual nutrients, foods, or food groups and their impact on health outcomes. It is likely that this was related to the earlier focus on nutrient-specific deficiencies [18]. However, as individuals consume foods and beverages in various combinations, there has been a shift in epidemiological studies toward investigating dietary patterns [19-21]. A dietary pattern can be described as the typical quantities, proportions, variety, or combination of foods and drinks that an individual consumes. The dietary pattern analysis approach offers advantages, as nutrients present in food can confound or interact with each other. In addition, pattern analysis can enable the detection of associations between diet and health outcomes because the combined effect of an entire diet may be more powerful than the effects of its individual components [20]. However, identifying data-driven dietary patterns relies heavily on subjective and arbitrary decisions regarding the grouping of food items and the labeling of dietary patterns. Previous studies have mainly derived dietary patterns from self-reported data [22]. In this study, our hypothesis is that by using tensorial PCA, we will be able to explore food purchase patterns from loyalty card data more comprehensively than before. This presumption is based on the ability to simultaneously detect patterns in both the product group dimension and the temporal trajectory enabled by tensorial PCA, yielding a deeper understanding of product group preferences, purchasing behavior, and temporal patterns. Tensorial PCA has been used in various data domains such as functional magnetic resonance imaging (fMRI) data [23], image and facial recognition [24,25], videos [26], and financial time series prediction [27]; however, our study is the first to focus on retailer data.

Thus, we provide a case study on tensorial PCA that allows us to identify temporal characteristics of health-related purchase behavior from longitudinal loyalty card (LoCard) data. By implementing modern tensorial variants of PCA on high-resolution data, we systematically searched for typical food purchasing patterns across time and product groups, atypical behavior across product groups and time, and differing purchasing behaviors across groups of cardholders. We showcase and discuss the interpretation, benefits, and challenges of these methods compared with their standard counterparts.

This study contributes to the interdisciplinary fields of biostatistics, human nutrition, public health, and digital health data science. The overall purpose of this methodological study is to show how tensorial PCA enables a comprehensive analysis; to demonstrate how this analysis can be used for retailer data; and, subsequently, to interpret the findings of dietary pattern analysis.

## Methods

### Materials: LoCard Data

#### Setting and Participants

Loyalty card data by the S Group, a major Finnish retailer cooperative, disclosed the background characteristics of the consenting card holders (age, gender, and residential postal code) and grocery expenditure data from the year 2016. Consenting card holders were from Helsinki and 9 nearby municipalities. The full details of the data collection process are described by Nevalainen et al [2]. The initial data on expenditure consisted of the purchases of 143 product groups of 14,595 consenting loyalty card holders, who were the primary card holders of their households.

#### Exclusion Criteria

To analyze the purchase behavior of regular customers, we excluded personnel members (1962 card holders) and card holders who appeared to be frequently absent or to conduct a substantial amount of their grocery purchases elsewhere, deﬁned as having: (1) >8 weeks, approximately 15% of all weeks, with no registered purchases at all (5919 card holders excluded) or (2) total expenditure of <€500 (US $547.3) per year (1331 card holders excluded). On average, a Finnish household annually used €4381 (US $4796) [28]; thus, €500 (US $547.3) is >10% of the average annual food expenditure and approximately 10 euros per week. Some of the card holders were excluded owing to more than one criterion, and after all exclusions, the final data set comprised 7251 card holders. The distributions of the demographic variables of those included and excluded were similar (Figure S1 in Multimedia Appendix 1 [23,24,29,30]).

#### Expenditure Variables

The initial grouping of foods provided by the retailer was based not only on ingredients but also on the package form and placement in the shelf system in stores. A nutrition researcher then regrouped the food, tobacco, and alcohol product groups into 76 groups relevant to nutrition and health research. Finally, product groups with minimal importance and expenditure, such as meal ingredients, were omitted, and the final data comprised 55 scientifically interpretable food, tobacco, and alcohol product groups. We used several criteria in our regrouping process: food price and status, connections to lifestyle or life stage, occasion of use, and wholesomeness. For instance, Tex-mex products were kept as a separate group because their consumption may be associated with special occasions such as parties or social evenings. The food group variables were comprehensive and nonoverlapping [10]. The variables were initially expressed in expenditure, that is, in euros spent on them. To give equal weighting to every card holder and to aid the interpretation of expenditure, we rescaled the expenditures in such a way that the annual expenditure for each card holder sums up to €1000. Thus, for each card holder, we observed standardized expenditure variables measured weekly, which can be interpreted as how many euros out of the €1000 were spent on product groups such as tobacco, vegetables, and meat. This scaling was necessary because the available data pertain to households of different sizes, structures, and shares of purchases from S Group rather than individual food consumption. By rescaling the expenditures, we enabled meaningful comparisons and analysis of purchase composition across different card holders. The resulting dimension of the data tensor was 7251×55×52 (card holders, product groups, and weeks). The product groups and their labels are presented in Table S1 in Multimedia Appendix 1.

### Statistical Methods

#### Principal Component Analysis

PCA has become a standard multivariate tool in dimension reduction [29,31]. With PCA, the aim is to reduce the original number of dimensions (ie, p) to a smaller number of derived variables (ie, k) that are linear combinations of the original variables such that “no information is lost” in terms of preserving as much of the variation as possible.

#### PCA for 3D Data

To provide a contrast to our proposed approach, we briefly expand on how PCA can be used to extract latent information from a horizontal cross-section of the LoCard data. Let the data matrix of **X** ∈ **R**^*n*×*p*^ contain the aggregated purchase data of *n* customers over *p* items during, for example, a single fixed week. The column-centered data matrix is denoted by 
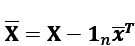
, where **1**_*n*_∈ **R**^*n*^ is a vector full of ones, and 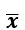 contains the means of *p* items over *n* customers. Let the columns of **U** ∈ **R**^*p*×*k*^ contain the eigenvectors of the sample covariance matrix



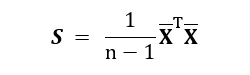



associated with its *k* (a user-chosen parameter) largest eigenvalues. The *k* columns of 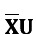 are known as the principal components, and they are usually expected to contain “hidden” information that is not discernible in the visualization of the original data. Moreover, the columns of **U** (“loadings”) can be used to interpret the components in terms of the original *p* variables. This type of PCA analysis could be undertaken for the LoCard data for a time-aggregated version of the data or for a single time occasion.

However, the full data are expressed as a 3D object **X** ∈ **R**^*n*×*p*×*t*^, where the additional dimension corresponds to the *t* time points during which the purchase history of each customer is recorded. The total data set (or data tensor) is most conveniently visualized as longitudinal data with a data matrix, **Y**_*i*_=**X**_*i*_,.,.∈ **R**^*p*×*t*^, *i*=1,...,n of purchase history associated with each of the *n* customers. A standard way of applying PCA to data with such a structure is to vectorize the matrices **Y**_*i*_ into long vectors *vec*(**Y**_*i*_) ∈ **R**^*pt*^ by stacking their columns. PCA can then be applied to the vectorization data matrix (*vec*(**Y**_*i*_),...,*vec*(**Y**_*n*_))^T^. However, this approach compromises the data structure by mixing the time and item dimensions, making the interpretation of the resulting principal components needlessly complicated. For example, in our data, having 55 product groups purchased at 52 weeks by customers would mean that the loading vectors have 2860 elements to interpret, making it very difficult to understand what they represent.

#### Structure-Preserving Dimension Reduction

A more preferable goal is to keep the 2 dimensions, product groups and time, separate and reduce their sizes individually. This approach is adopted in *tensorial dimension reduction*, a methodology aimed at reducing the dimensionalities of data sets consisting of observations of higher order than ordinary vectors such as matrices [32]. An extension of PCA in this case is higher-order singular value decomposition, known as higher-order singular value decomposition [30] and later rediscovered under the names 2-directional 2-dimensional PCA [24] and tensorial PCA [23], depending on the context, which also begins by centering the observed item-time matrices over the following sample:



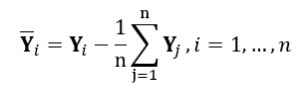



The next step is to compute the *modal covariance matrices* of product groups and time separately, as follows:



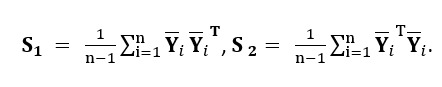



The interpretation of **S**_1_ is that it measures linear dependency among items while ignoring (or aggregating over) the time space, and vice versa for **S**_2_. To obtain the principal components, we consider the first *p*_0_ eigenvectors (principal item directions) 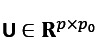 of **S**_1_ and the first *t*_0_ eigenvectors (principal time directions) 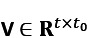 of **S**_2_, where *p* is the number of product groups, *t* is the number of weeks, and *p*_0_ and *t*_0_ are user-specified parameters for the number of principal components to be selected for further analysis. To aid in choosing *p*_0_ and *t*_0_, the eigenvalues of **S**_1_ and **S**_2_ can be plotted as in PCA to obtain scree plots. In such scree plots, one assumes that the last *p − p*_0_ and *t − t*_0_ eigenvalues are equal and one searches an “elbow.” To aid the choice, information on eigenvector variation can be incorporated using resampling methods [33,34], where, for example, in the augmentation approach, the idea is to augment the data tensor mode wise. The criterion is then a weighted sum of the eigenvalues and eigenvector variation [35,36]. Here, we have used this augmentation method for selecting the suitable number of principal components for further analysis. The augmentation estimator is a nonheuristic method that guarantees the ability to estimate the dimension correctly under mild conditions [36].

The individual matrices of the principal components for each customer are then obtained as projections 
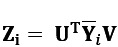
. We note that although the dimension reduction is performed separately for the item and time spaces, the method still produces a single matrix of principal components for each customer, in which each component is related to a time-space direction pair. An example of interpreting the tensorial PCA components and directions is provided in Multimedia Appendix 1 (Interpretation of the Tensorial PCA Components and Directions).

The main assumption of tensorial PCA is that the variation in data can be decomposed into row and column variations. Typically, this is expressed mathematically by requiring that the observed matrices **Y**_*i*_ admit representations **Y**_*i*_=**A** **Z**_*i*_ **B**^T^, where the latent matrix **Z**_i_ has uncorrelated components, and **A** and **B** are orthogonal matrices of suitable sizes [36].

#### Interpretation of the Scores and Loadings Resulting From Tensorial PCA

Tensorial PCA, similar to standard PCA, transforms data from its original coordinates (variables) into a new coordinate system, where each coordinate, called principal component scores, corresponds to a linear combination of the original variables weighted by the estimated loadings. The scores are the new set of coordinates, where the first principal component captures most of the variability in the data, followed by the second principal component, and so on. Tensorial PCA returns these scores for each product group and week pair. In addition, tensorial PCA provides loadings representing the associations between each product group and the principal component (and each week and the principal component), reflecting the covariance between the component scores and the observations. When interpreting the loadings, the emphasis is on the magnitude of each loading. Variables with larger absolute loadings have a stronger influence on the principal component. The sign of the loading merely indicates the direction of the relationship (positive or negative correlation) between the variable and the component, both being equally interesting.

#### Illustration and Software Packages

To enhance the recognition of product groups and weeks exhibiting similar patterns, we used heat maps and hierarchical clustering of loadings (with correlation distance and average linkage) to illustrate purchase patterns. Hierarchical clustering is a standard clustering method that facilitates the visualization of a large number of loadings [37]. All analyses were performed with R (version 4.0.1; R Core Team) [38] and using packages *tensorBSS* [39], *gplots* [40], and *ggplot2* [41].

### Ethical Considerations

This study was approved by the University of Helsinki Review Board in the Humanities and Social and Behavioral Sciences (statement 43/2016). Before inclusion in the study, all participants were invited to participate via email and provided informed consent electronically. They were asked to release their loyalty card data. To ensure privacy, the data were pseudonymized by S Group before the researchers obtained the data.

## Results

### Descriptive Analysis of the Data

**Figure 1:**
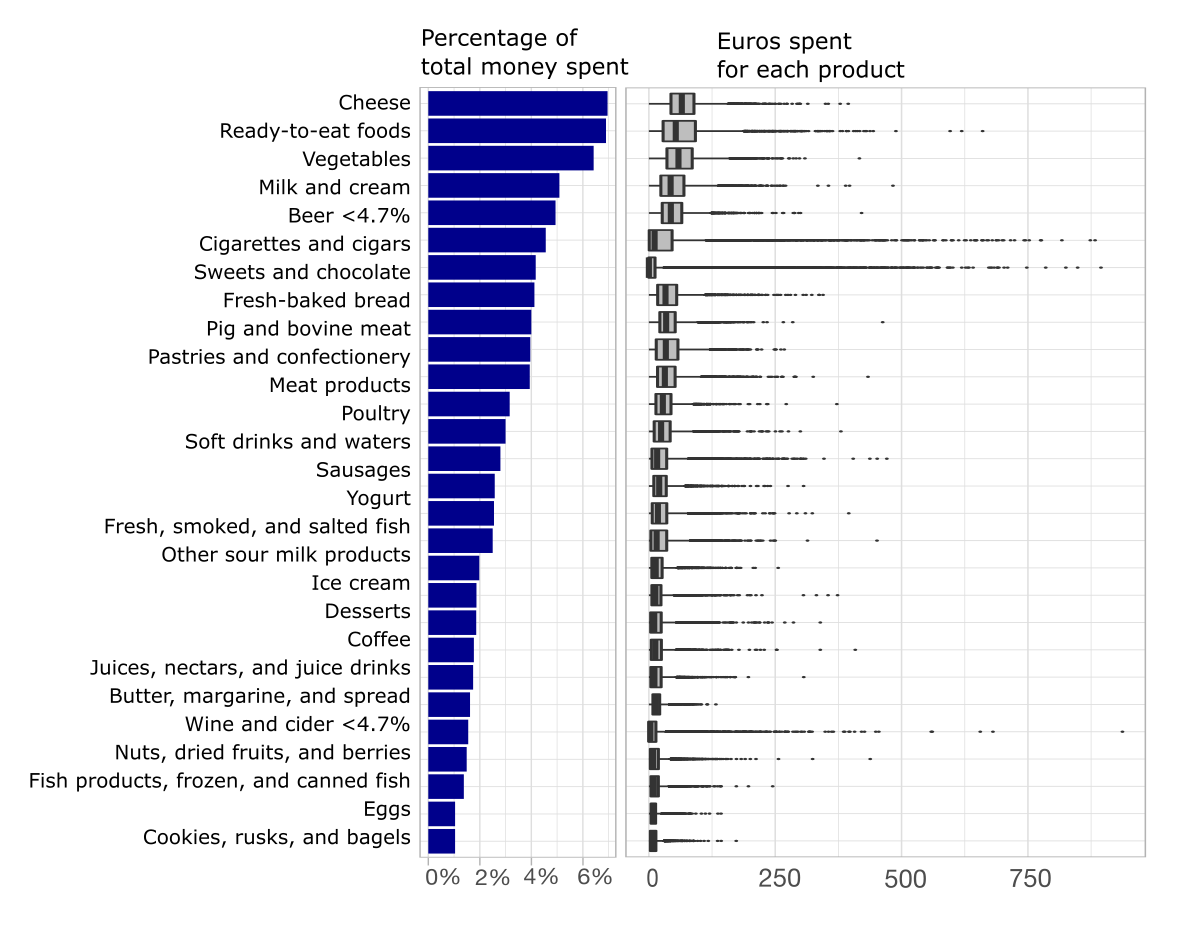
Money spent (per €1000) by product group across customers and average purchase basket of participants (y-axis is the percentage of money spent on each product group). Only the product groups covering >1% of all purchases are illustrated. Thus, altogether 27 product groups, that is, 11.01% of all purchases were omitted.

We begin by reporting simple summaries and illustrations of the raw rata. When considering the product groups, the highest median expenditure was for cheese, which constituted together 7% of all food purchases. The customer-wise maximum expenditures were on beer (€882 [US $961.4]), cigarettes (€894 [US $974.5]), and wine and cider (€937 [US $1021.3]), and for all customers combined, of all purchases, these product groups constituted shares of 4.6%, 4.2%, and 1.5%, respectively (Figure 1; Table S1 in Multimedia Appendix 1). In addition, purchases of many product groups, such as cigarettes and alcohol, increased for some customers, whereas many others did not purchase these items at all (Figure 1; Table S1 in Multimedia Appendix 1).

**Figure 2:**
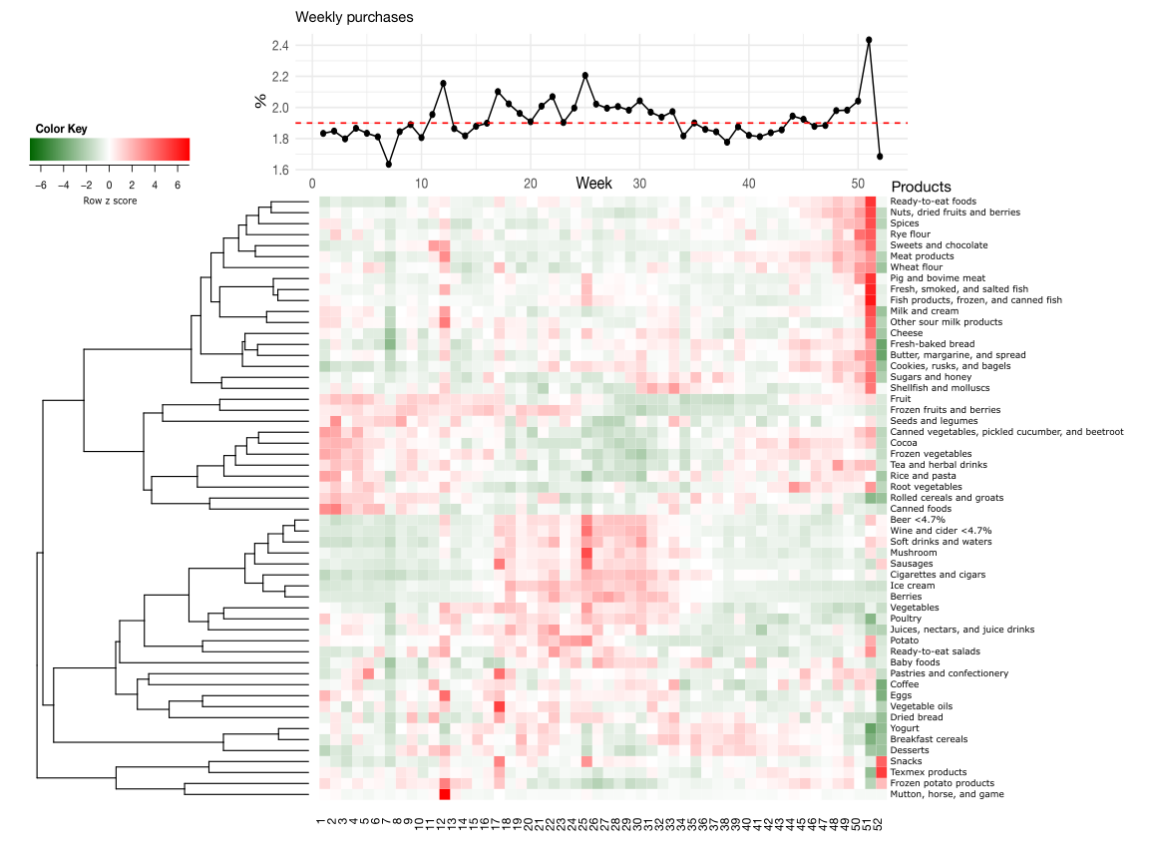
The upper line graph illustrates the percentage of purchases made across the weeks. Dashed red line represents 1.9%, which would be the weekly average if all purchases were distributed evenly across the year. Heat map shows weekly purchase pattern illustrated for total sum of the money spent on each product group (rows). The color indicates the row-wise z scores of each product group. Holiday weeks 12, 25, and 51 clearly stand out from the analysis. Simultaneously, the figure illustrates the sum patterns for the product groups, showing that some of the product groups are more often purchased during summer (eg, beer, wine, and cider), whereas others are purchased more during winter (eg, frozen fruits and frozen vegetables). Summed data are clustered with correlation distance and average linkage.

There were also obvious differences in the purchase patterns based on time. If all purchases were distributed evenly across weeks, then each week would constitute 1.9% of the yearly purchases. However, exceptional weeks, such as week 51 (Christmas), constituted 2.4% of all purchases, which was 26% more than the average week. Similarly, weeks 25 (Midsummer) and 12 (Easter) constituted 2.2% each, which was 16% more than purchases in an average week (Figure 2).

In addition, some of the product groups had a pattern that was related to the time of the year (Figure 2). For many product groups, the pattern was steady or there was only little weekly variation in the purchase behavior of customers. For some product groups, there were clear seasonal patterns during summer or winter and holiday times, that is, Christmas, Midsummer, and Easter, which can easily be distinguished from the weekly plot (Figure 2). For instance, the expenditure on beer tended to rise toward the summer season compared with other times of the year and to peak at Midsummer. Sweets and chocolate purchases clearly increased during Christmas and Easter, and pig and bovine meat had a similar trend, whereas mutton purchases increased clearly only during Easter.

### Joint Analysis of Time and Food Purchase Patterns

Using descriptive statistics and cluster analysis of product group expenditures over time, we were able to detect general patterns in the data. By using tensorial PCA, we could learn more from the data by taking the product group and time information into account simultaneously and could gain more insight into interindividual differences.

First, we analyzed the correlation structure of the product groups and time in Multimedia Appendix 1 (Correlation Structures of Products and Time). Especially weeks next to each other correlated heavily, which is a sign of serial correlation (Figure S2 in Multimedia Appendix 1). At the same time, the holiday weeks of Christmas (week 51), Easter (week 12), and Midsummer (week 25) stand out because of their understandably different purchase behavior; these may include different product groups and amounts compared with everyday life.

Tensorial PCA also allows outlier detection to be conducted simultaneously based on several dimensions [23,24,30]. Here, we used it for identifying atypicalities within the data for both time and product group dimensions, Multimedia Appendix 1 (Detecting Atypicalities). We revealed outstanding patterns during different seasons and holiday times, that is, weeks 12, 25, and 51, as well as patterns indicating dominant product groups, that is, beer, cigarettes, and wine and cider (Figure S3 in Multimedia Appendix 1).

Longer-term health behavior is generally more relevant than occasional behavior during holidays or other special occasions. Therefore, we wanted to assess longer-term health behavior by purchase patterns, focusing on periods other than the holiday seasons. The health risks of beer and alcohol have been well documented [42-45], and we felt that here the focus on food purchasing behavior is more insightful without their inclusion. Therefore, to analyze the purchase (dietary) patterns of everyday life, we peeled outlying data from the data entity by removing the holiday weeks as well as the beer, cigarette, and wine and cider product groups. With these filtered data, we could identify different types of purchase behavior patterns and detect groups of individuals whose purchase patterns stand out with the specific combination of time and product group.

**Figure 3:**
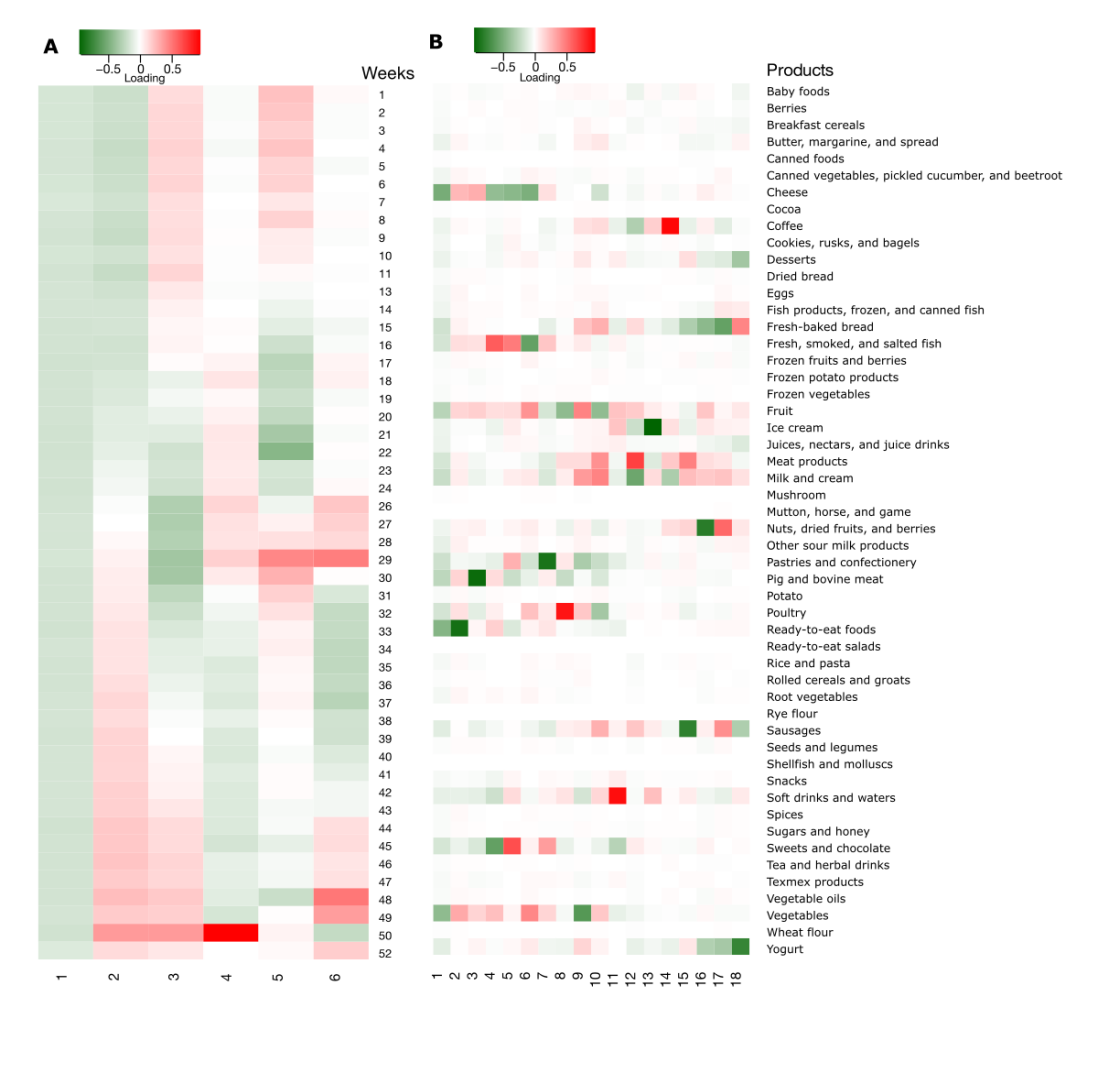
Loadings of tensorial principal component analysis components (x-axis) for (A) weeks and (B) product groups. Red indicates a high positive loading, and green indicates a high negative loading, both equally interesting.

After filtering out the holiday weeks 12, 25, and 51 as well as the purchases of beer, cigarettes, and wine and cider of the scaled data, the resulting new tensor of data is **X** ∈ **R**^7251×52×49^. These data are now free of the most obvious outliers in the time and product group dimensions and were used to analyze time trends and food purchase patterns. On the basis of the reanalysis with tensorial PCA and using the augmentation method, we identified 18 product group principal components, explaining 81.8% of the product group variation, and 6 principal components for the time dimension, explaining 34.7% of the time variation (Figure 3; Figure S4 in Multimedia Appendix 1).

Overall, tensorial PCA of the filtered data was easier to read. The first week-based principal component shows the average purchase pattern over the year (Figure 3A). This illustrates the average food expenditure per week, meaning that the largest source of variation for the week dimension is the general level of expenditure. The second component illustrates cases in which the purchase pattern differs between the first and second half of the year. This can be the case, for example, if a card holder encounters major changes in household structure (eg, moving in with someone or new family), has moved to another address, implemented a lifestyle change, or just changed the grocery store where most purchases were made. The third principal component shows the differential pattern of purchases during winter and summer. We refer to these as the first 3 time components: PC1—weekly average, PC2—spring versus autumn, and PC3—summer versus winter. The remaining components seem to be more detailed variants of these, indicating, for instance, school or work holidays (PC4).

On the basis of the product group components, the first component gave high loadings for multiple product groups, with most of the product groups being fairly regular items in a Finnish food basket (Figure 3B). The other components were more product group specific. The second component loads highly on ready-to-eat food (including a variety of packaged and service counters selling ready meal portions such as pasta and pizza) and contrasts it with several fresh product groups such as pig and bovine meat, vegetables, and fruits. Therefore, with the second component, we can identify card holders who buy substantial amounts of ready-to-eat food but purchase very few fresh product groups and simultaneously card holders who buy only very little ready-to-eat food and a lot of fresh product groups. Thus, with each component, we can simultaneously detect the customers at both ends. The third component loads highly for pig and bovine meat, indicating a strong preference for eating inexpensive meats. We refer to these as the first 3 product group components: PC1—product average, PC2—ready-to-eat, and PC3—red meat.

**Figure 4:**
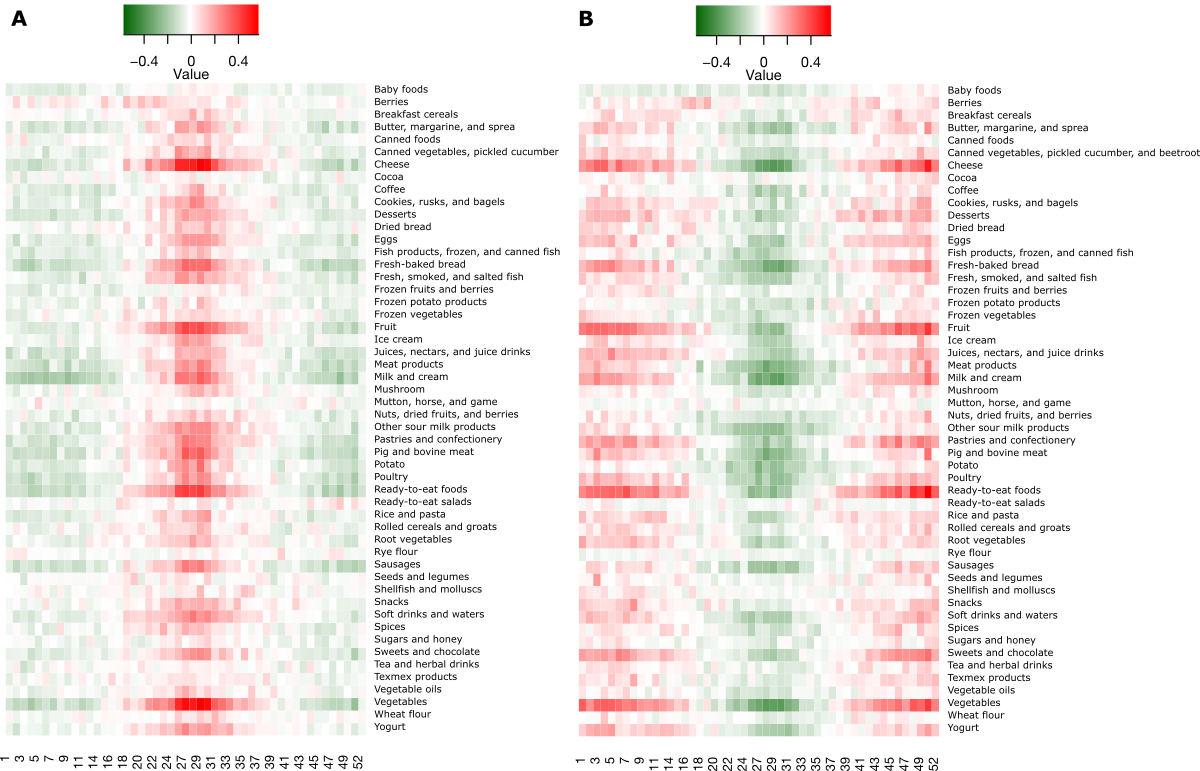
Illustration of the average purchase pattern of the card owners having the highest (A) and lowest (B) 10% scores of the third principal component of weeks (PC3—summer vs winter) and the first principal component of product groups (PC1—product average). The values have been standardized against all customers, after which the averages were computed, that is, a dark green value indicates that the average money spent within the illustrated group on each product group is 0.4 SDs lower than the average across all customers and a strong red value indicates that the average money spent within the illustrated group on each product group is 0.4 SDs higher than the average across all customers.

Using these loadings, we simultaneously selected the time pattern and the product group pattern to illustrate individuals who exhibit such purchase patterns. We categorized our subjects into 3 categories: the 10% with the highest scores of the principal components formed the group “high” and the 10% with the lowest scores of the principal components formed the group “low,” with 80% with scores between these 2 extremes representing the group “typical.” This was done separately for the scores of each pair of the week and product group. In Figure 4, we illustrate the purchase pattern of the high and low groups defined by the scores of the principal components for the combination of week component 3, that is, PC3—summer versus winter and product group component 1 PC1–weekly average. As the first product group component takes the “average” and the third week component contrasts summer with winter, the seasonality of the highest and lowest deciles is clear; the figures reveal the juxtaposition between summer and winter observed in the loadings for the third time component. Moreover, this pattern persists across almost all product groups, which is in line with the first principal component of product groups representing an approximated average over a large number of product groups.

In addition, we selected the product groups with an absolute loading >0.3 in at least 1 of the selected 18 components and illustrated their purchase patterns for the groups high and low defined by all combinations of the first 3 principal components of weeks and the product groups (Figure S5 in Multimedia Appendix 1). The first product group based on the principal component shows the average expenditure of each group (PC1—product average), whereas the second focuses on pig and bovine meat (PC2—red meat) and the third on the tendency to buy ready-to-eat foods (PC3—ready-to-eat). These illustrations demonstrate how tensorial PCA identifies individuals with specific purchase patterns.

**Figure 5:**
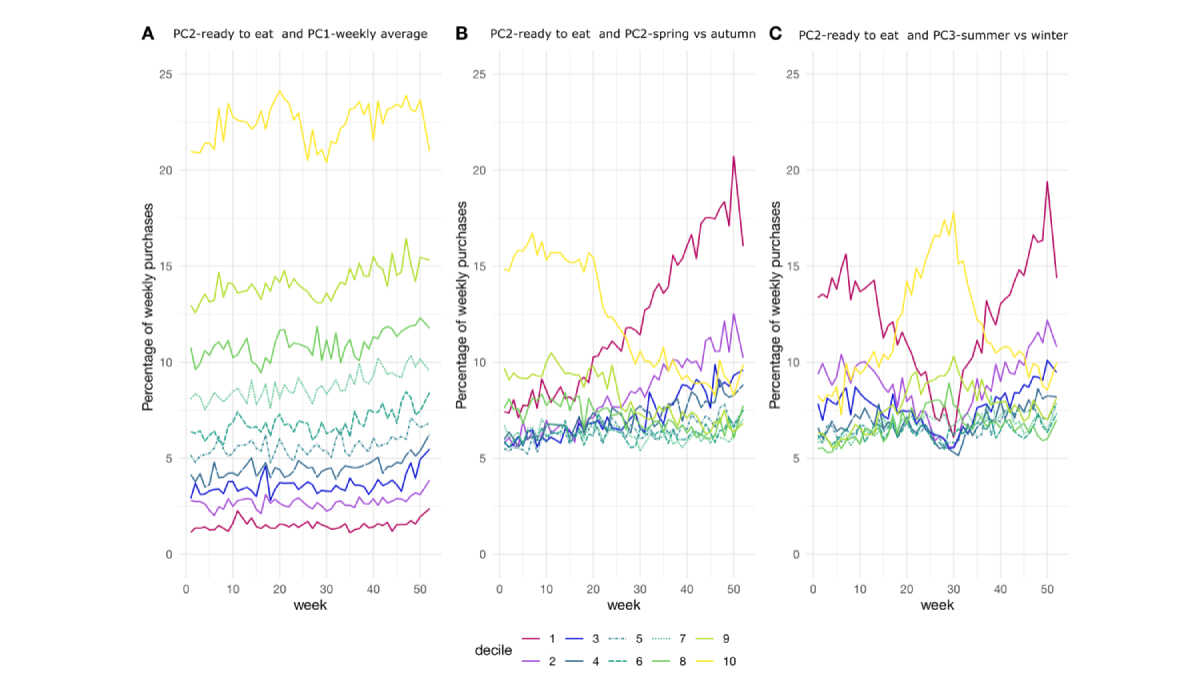
Average percentages of weekly expenditures on ready-to-eat foods, with the deciles divided based on the product group PC2-ready-to-eat and time PC1-weekly average (A), time PC2-spring versus autumn (B), and time PC3-summer versus winter (C). (A) The first time component finds the groups of participants with different levels but temporally stable purchase behavior for ready-to-eat food. (B) The second time component and especially its extreme deciles reveal the participants with increased or decreased use of ready-to-eat food. (C) With the third time component, we can identify participants with seasonal change in the ready-to-eat food purchase pattern.

**Figure 6:**
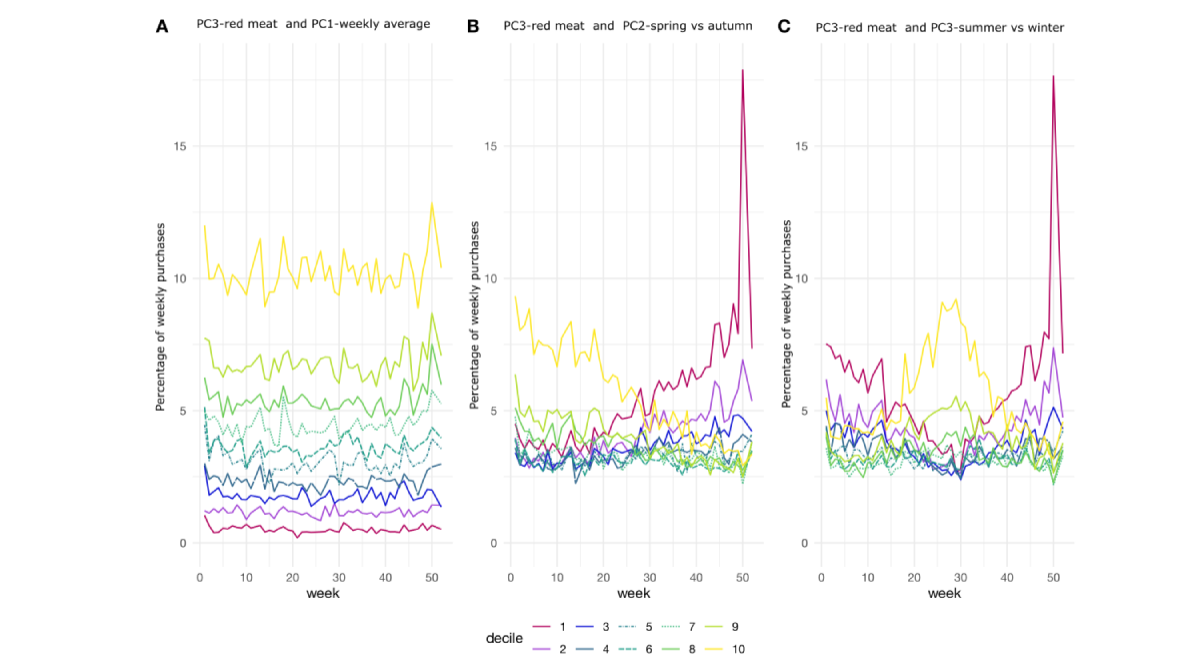
Average percentages of weekly expenditures on red meat with the deciles divided based on the product group PC3-red meat and time PC1-weekly average (A), time PC2-spring versus autumn (B), and time PC3-summer versus winter (C). (A) The first time component finds the groups of participants with different levels but temporally stable purchase behavior for red meat. (B) The second time component reveals the participants with increased or decreased meat, and (C) detects the participants with summer versus winter difference in meat purchases.

Furthermore, as ready-to-eat foods and pig and bovine meat (hereafter referred to as red meat) were clearly detected in principal components 2 and 3, we focused on them specifically. We divided the participants into deciles based on the selected component and week scores and illustrated them in Figures 5 and 6, in which we can clearly identify the different purchase patterns for the product groups throughout the weeks. For example, in Figures 5A and 6A, we can see the participants with steady differential purchase preferences of the product groups, whereas in Figures 5B and 6B, we see participants with consistently evolving changes in their purchase patterns. In addition, Figures 5C and 6C illustrate the groups having different seasonal purchases of the product groups in winter and summer. For the first deciles of red meat and PC2—spring versus autumn as well as PC3—summer versus winter (Figures 6B and 6C), the week before Christmas still spiked clearly: it is the time of year when Finnish people purchase ham for the Christmas table.

These values are the percentages of weekly purchases, reflecting the proportion of ready-to-eat foods or red meat out of the total purchases. Thus, the percentages indicate changes in the relative proportion of these categories and not changes in total expenditure. In addition, note that, for example, the highest decile in panel A consists of individuals different from those in panel B. This means that we could in principle correlate the scores of each panel with other variables, and potentially clarify the reasons for or the loyalty card holders behind the stable or changing purchase behavior.

### Comparison With the Results of Standard PCA

Table 1 summarizes the differences between the tensorial PCA and standard PCA results. The most evident difference in analyzing the data is that the format of the input data is different. Although tensorial PCA input data are multidimensional **X** ∈ **R**^7251×52×49^, the data input to standard PCA is in a 2D matrix format.

To compare the actual results for purchases, we ran the standard PCA for the same filtered data and compared the results with those of tensorial PCA (Table 1). We ran the standard PCA with three data sets modified from the tensor data: (1) *product group data:* data were summed to the amount of money spent on each product group throughout the year **X** ∈ **R**^7251×52^; (2) *weekly data:* data were summed to the money spent in each week **X** ∈ **R**^7251×49^; and (3) *combination of product group and week*: combines all information in the tensor as such to a matrix **X** ∈ **R**^7251×(52*49)^=**R**^7251×2548^, where every week-product group combination has its own column.

Using the augmentation method for standard PCA, we selected 22 out of 52 product group components, 4 out of 49 week components, and 40 out of 2548 components for further analysis (Table 1). The analysis revealed that most of the variation between the participants came from the product groups that they buy, rather than from the purchase times, as the loadings behaved very similarly across the weeks within the product groups (Figures S6 and S7 in Multimedia Appendix 1). These results are also consistent with earlier results of tensorial PCA showing very similar patterns of PC loadings as well as weekly loadings that are smaller than the loadings of the product groups (Figure 3; Figure S4 in Multimedia Appendix 1).

In addition, to compare the results of the standard PCA and tensorial PCA, we computed correlations between the scores of participants based on the first component and (1,1) component of tensorial PCA (PC1—weekly average and PC1—product average), yielding a significant correlation between the product group-wise scores, reaching *r*=0.988 over the first few components (Figure S8 in Multimedia Appendix 1). Therefore, the standard PCA for the aggregated data yielded approximately the same results as the tensorial PCA for the product group components combined with the first time PC. Recall that the first time component constructed approximately “an average over the year” (PC1—weekly average), which led, not surprisingly, to practically the same result. This finding was also observed for the PC2—ready-to-eat food and PC3—red meat component scores, with the first PC1—weekly average (Figure S8 in Multimedia Appendix 1). However, although the standard PCA could detect the time pattern of the summed purchases in week-based data, it could not easily do this for product groups. Tensorial PCA allowed us to delve deeper into the data and detect the most important changes in patterns over time for the most important product group combinations, a property that is potentially very useful for high time resolution. With standard PCA, we could also combine the product groups and weeks as separate variables; however, as the product group variation was much higher than the variation between weeks, it was not possible to detect timewise variation in the results (Figure S6 in Multimedia Appendix 1).

**Table 1:** Comparison of standard and tensorial principal component analysis (PCA) of the same but differently arranged data.

Characteristic	PCA for product group data	PCA for weekly data	PCA for product group+week combinations	Tensorial PCA
Interpretation of the results	“Money spent in total on each product group”	“Money spent in total in each week”	“Money spent on each product group in each week in 2D format”	“Money spent on each product group in each week in 3D format”
Data size	**X** ∈ **R**^7251×52^	**X** ∈ **R**^7251×49^	**X** ∈ **R**^7251×2548^	**X** ∈ **R**^7251×52×49^
First dimension	Participants	Participants	Participants	Participants
Second dimension	Product groups	Weeks	Product groups×weeks	Product groups
Third dimension	N/A^a^	N/A	N/A	Weeks
Number of significant principal components based on the augmentation method	Product groups: 22	Weeks: 4	Combinations of product groups and weeks: 40	Product groups: 18 and weeks: 6
Cumulative percentage of explained variation	Product groups: 59.5%	Weeks: 14.5%	Combinations of product groups and weeks: 19.4%	Product groups: 81.8%; week: 34.7%
Can find purchase pattern in product groups	Yes	No	Yes	Yes
Can find purchase patterns across time	No	Yes	Yes, but patterns of product groups dominate patterns of times	Yes
Was successful in finding product group pattern–specific yearly trends	No	No	No	Yes
Notes in interpretation	Easy to interpret and limited insight owing to aggregation of time dimension	Easy to interpret and limited insight owing to product group aggregation	Difficult to interpret as loadings are for combinations of product groups and weeks	Both product group and week need to be taken into account in interpretation

^a^N/A: not applicable.

## Discussion

### Principal Findings

Loyalty card data, an automatic recording of all grocery purchases of the card owner, can be used to analyze the health behavior of customers [46]. Continuous data collection provides not only product group-wise information but also a time component showing when purchases were made. With tensorial PCA, we were able to analyze this multidimensional purchase data in greater detail than before by simultaneously focusing on both time and product group dimensions. The key advantage of tensorial PCA over standard PCA is its ability to effectively capture changes in patterns over time for specific product combinations. Although standard PCA detected the overall time pattern of the summed purchases in our week-based data, it faced challenges in detecting the temporal pattern when observations on different weeks and product groups were expressed as vectors (Figure S6C in Multimedia Appendix 1). Tensorial PCA, in contrast, allowed us to analyze not only at the aggregate level but also deeper; we were able to uncover the most significant changes in patterns over time and product groups. By leveraging the tensor structure of the data, tensorial PCA effectively captured the interplay between products and periods, enabling us to identify temporal variations in product-specific purchasing behaviors. It provided valuable insights into the dynamics of product combinations, allowing us to identify temporal shifts in card holders’ purchasing patterns more accurately. This characteristic could be important in, for instance, assessing changing dietary (purchase) patterns following external alterations, such as price change following a price inflation or policy implementation (eg, sugar tax) or major disruptions in society (eg, lockdown during a pandemic).

### Comparison With Prior Work

Traditional PCA has been widely used for nutrition data among different settings, cultures, and sociodemographic groups. Although the naming of the patterns and the foods loading to the components may vary slightly, typically at least 2 common patterns are identified in most countries, as in Finland: a prudent, healthy dietary pattern and an unhealthy “Western” pattern [47-51]. A third, almost equally typical pattern is often termed “traditional” [52], and this pattern is generally more context specific. A traditional Finnish diet is characterized by sausages, potatoes, milk, coffee, and butter [53]. A ready-to-eat pattern has also often been identified, characterized by a high consumption of ready-to-eat meals [51,54]. Similarly, a pattern indicating alcohol consumption has been identified previously in Finland [55]. As mentioned in our results, the standard PCA for the aggregated data yielded similar results to the tensorial PCA for the product group components combined with the first time PC. The added value of tensorial PCA was that it enabled us to identify patterns indicative of broader trends in purchasing behavior and to identify specific weeks for specific product groups.

The use of grocery purchase data for health research purposes is still relatively novel, and, to the best of our knowledge, only a few earlier studies have identified dietary patterns based on customer loyalty card data. One such study was conducted in the United Kingdom [3], whereas the other is an earlier study conducted by our group [51]. Both studies used the standard PCA method for analysis. Our previous study identified 8 patterns based on a more detailed food grouping within the same LoCard data used in this study [53]. Consistent with previous findings, we also identified patterns characterized by a high consumption of ready-to-eat products and red meat. Moreover, barcode scanning was used to examine purchase patterns, revealing a consistent finding of a pattern characterized by high consumption of ready-to-eat meals [54]. A direct comparison between our study and earlier studies on purchase patterns is not feasible because of several reasons. First, different countries have distinct food cultures that can significantly impact the identified patterns. In addition, the analyzed food product groups may consist of different items across studies, further complicating direct comparisons. Finally, the choice of analytic methods used can introduce differences in the results obtained. Therefore, it is important to acknowledge these issues and approach the comparison with caution.

### Strengths and Limitations

The main strength of this study is the vast data set used, as S Group is a leading retailer with a market share as high as 47.2% in Finland [56]. Its shops cover the entire country, thus providing an excellent means of investigating the purchase patterns of Finnish people on a large scale. With grocery stores constantly collecting more detailed data and, thus, also loyalty card data having more dimensions than ever before, tensorial PCA provides an optimal means to analyze such data. Tensorial PCA simultaneously focuses on multiple dimensions and finds principal components in a dimension that otherwise would have been masked by another dimension with higher variability. Some limitations of this study also need to be addressed. First, there are other grocery stores in Finland; therefore, not all grocery purchases of the households are included. Second, only transactions made by customers using their loyalty card are captured. Although there are significant benefits for customers associated with using the card, not all customers actually use or even carry it with them, and the results thus perhaps represent the customers of S Group rather than the Finnish adult population. However, we have previously found that the age distribution of participants of the LoCard study is similar to that of the residents within the region, although the proportion of women is higher among loyalty customers (67.3%) than among residents (52.1%) [2]. Therefore, the interpretation of data is constrained by these limitations [57]. It should also be noted that the naming of PCA-derived purchase patterns is a highly subjective decision. Therefore, it is of great importance to publish information on food grouping and factor loadings along with a thoughtful naming of the components. This will enable the assessment of reproducibility and similarity.

### Future Work

The purchase patterns disclosed here can be used in subsequent analysis. For example, we can identify the use of meat product groups and determine whether it is related to specific times of the year or whether it is stable throughout the year. In this way, the impact of timed social interventions, such as meat-free October or vegetarian months, on the purchase of meat can be evaluated. Several year trends in the consumption of red meat and its substitutes would also be important to monitor and understand [58,59].

We could determine the association between customer demographics (such as age and gender) and patterns to understand the behavior of different population subgroups, potentially important in sustainable food consumption, human health, and retailers’ interests. For example, we can identify the type of individuals who buy more meat during summer or whose purchase pattern is focused more on vegetables overall or at a specific time of the year. Sociodemographic studies show that educated urban women are ahead of the curve in moving toward a more sustainable diet [59-62]. They are better able to follow the path of a larger sustainable dietary change, whereas for some other population groups, such as men and those less educated, making smaller dietary changes is more likely to be successful [59,61]. Tensorial PCA can be used for detailed monitoring of the nutrition and vulnerability of different population groups in a food system transformation and, for example, when significant changes occur in food prices.

In addition, the analysis can be used to detect the effects of external factors, including inflation or a global crisis, such as the COVID-19 pandemic or the Ukraine war, on the everyday purchase behavior of customer groups. The persistence of these changes can also be evaluated, yielding insights into which sociodemographic groups are most affected by these external factors. Tensorial PCA will also help to identify the influence of national steering instruments, such as taxation and updated nutrition recommendations, on purchase trends over time. In problems such as these, the ideal data would be multidimensional and of high resolution for each dimension. This makes tensorial dimension reduction methods, such as tensorial PCA, valuable in these contexts, as they can both reduce the data dimension in an interpretable manner and keep the subject, product group, and time dimensions separate.

### Conclusions

This is the first study on the weekly purchase patterns of Finnish customers that simultaneously considers both the time and product group dimensions for each customer. With tensorial PCA, we could identify abnormalities in the purchasing behaviors during specific weeks or for specific product groups and were able to detect patterns of wider trends in customers’ purchasing behavior. By using standard PCA, we found the principal components for either the weeks or the product groups, whereas with tensorial PCA, we identified purchase patterns simultaneously based on the dimensions in the data tensor. By selecting specific features identified based on the patterns within each dimension, we detected the participant groups with specific purchase patterns. In further analyses, these patterns based on time and product groups are likely to be directly linked to the socioeconomic and demographic predictors of dietary patterns of customers. These associations will enable us to identify what types of people have specific purchasing patterns, which in turn can assist in the development of ways in which consumers can be steered toward making healthier food choices.

## Appendix

Multimedia Appendix 1 Supplementary text, tables, and figures.

## Abbreviations

fMRI: functional magnetic resonance imaging
PCA: principal component analysis

## Data Availability

The data used in this study are owned by a third party (S Group) and used under a research agreement. The data underlying this study cannot be shared publicly for the privacy of individuals who participated in the study.
