# Supplementary material for: Tensorial Principal Component Analysis in Detecting Temporal Trajectories of Purchase Patterns in Loyalty Card Data: Retrospective Cohort Study
Source: J Med Internet Res. 2023 Dec 15;25:e44599. doi: 10.2196/44599 (PMC10757224; doi:10.2196/44599)
Supplement: Multimedia Appendix 1 [file jmir_v25i1e44599_app1.pdf]

Supplement to the article: Tensorial Principal Component Analysis in Detecting Temporal Trajectories of Purchase Patterns in Loyalty Card Data: Retrospective Cohort Study

Reija Autio, Joni Virta, Klaus Nordhausen, Mikael Fogelholm, Maijaliisa Erkkola, and Jaakko Nevalainen

Interpretation of the Tensorial PCA Components and Directions

We next give examples of interpreting the tensorial PCA components and directions. Assume for simplicity that we have a total of p = 3 different items and t = 4 time points and that we have chosen to retain p_0 = 2 principal item directions and t_0 = 2 principal time directions (attaining a dimension reduction from pt = 12 elements to p_0t_0 = 4 elements). Let the directions be as follows:

$$\mathbf{U} = \begin{pmatrix} 1 & 0 \\ 0 & \frac{1}{\sqrt{2}} \\ 0 & -\frac{1}{\sqrt{2}} \end{pmatrix}, \quad \mathbf{V} = \begin{pmatrix} \frac{1}{2} & \frac{1}{2} \\ \frac{1}{2} & \frac{1}{2} \\ \frac{1}{2} & -\frac{1}{2} \\ \frac{1}{2} & -\frac{1}{2} \end{pmatrix}.$$

The principal components $\mathbf{Z} = \mathbf{U}^T\bar{\mathbf{Y}}\mathbf{V}$ (where we have dropped the index i for convenience) then take the form:

$$\mathbf{Z} = \begin{pmatrix} \frac{1}{2}y_{1,1:4} & \frac{1}{2}(y_{1,1:2} - y_{1,3:4}) \\ \frac{1}{2\sqrt{2}}(y_{2,1:4} - y_{3,1:4}) & \frac{1}{2\sqrt{2}}([y_{2,1:2} + y_{3,3:4}] - [y_{2,3:4} + y_{3,1:2}]) \end{pmatrix},$$

where $x_{k,s:t}$ denotes the sum of the elements on the kth row of $\mathbf{Y}$ over the columns from s to t, that is, the total (centered) purchase amount of the product k over the time points from s to t. The interpretations of the four components in $\mathbf{Z}$ are as follows:

- The (1,1) principal component measures (and gets large values for people who have large) overall purchase amount of item 1. This is evident in the direction matrices by $\mathbf{u}_1$, the first column of $\mathbf{U}$, being dominated by the first item and $\mathbf{v}_1$, the first column of $\mathbf{V}$, having spread evenly over all four time points. This kind of pattern can emerge if the data contain an item, e.g. bread, that is typically purchased by all customers throughout the year.

- The (1,2) principal component measures the contrast in the purchase amounts of item 1 during the first and last two time points. This can be seen from the direction $\mathbf{v}_2$ having elements of equal magnitude but opposite signs. This kind of pattern can emerge if the data contain a product, e.g. (non-frozen) berries, that is purchased less than average during a certain period of time and more than average during another period of time.
- The (2,1) principal component measures the contrast between the overall purchase amounts of items 2 and 3, i.e. someone with a large value for this component has purchased greater than average amounts of item 2 and less than average amounts of item 3. This kind of pattern can emerge if the data contain two items, e.g. vegetarian products and meat, that are typically not purchased together by the same customer.
- The (2,2) principal component is a combination of the previous two contrasts, i.e. someone with a large value for this component has purchased greater than average amounts of item 2 during the first two time points and item 3 during the last two time points, and vice versa. This kind of pattern can emerge if the data contain two products, e.g. ice cream and warm beverages, which are associated with two opposing time periods.

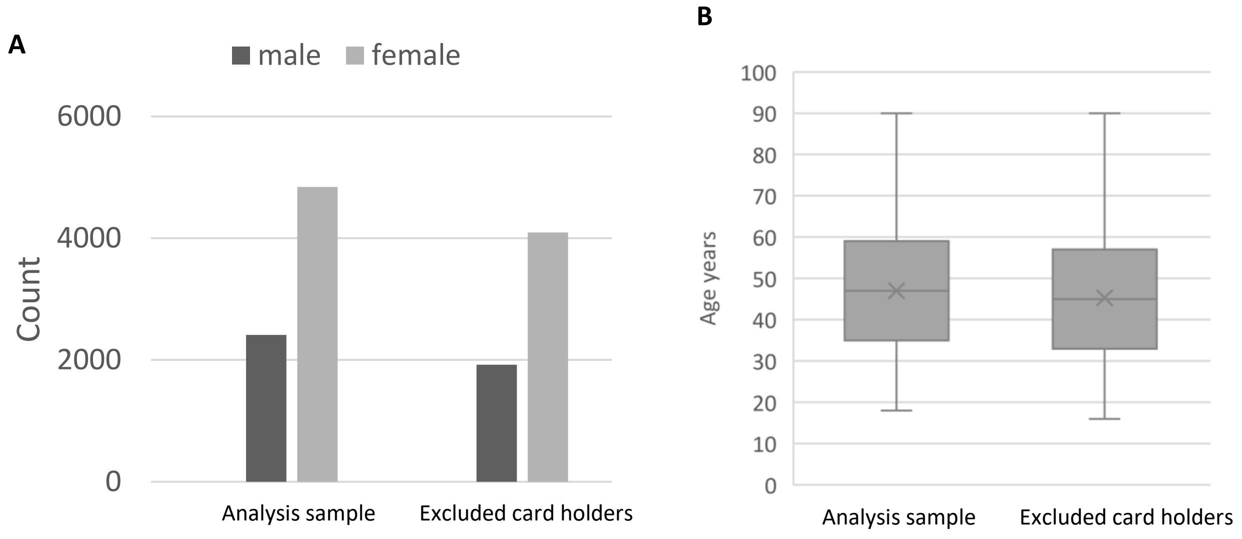

Figure S1: A) Comparison of the gender distribution of individuals in the study and excluded. Of the ones within study 66.7% were female, while of the excluded ones 68% were female. B) The mean age of the card holders within study was 47.0 years (SD 14.6) and in excluded 45.3 years (SD 14.7).

Table S1. Names of products and descriptives of data. Median, minimum, and maximum across all customers show the euro expenditure per each 1000€ spent per each product. The last column gives the percentages of the money all card users spent on the product.

Product	Median across all customers (€)	Min (€ of each 1000€)	Max (€ of each 1000€)	% of total money spent
**Baby foods**	0.00	0.00	439.85	0.60
**Beer <4.7%**	11.33	0.00	882.89	4.56
**Berries**	1.31	0.00	226.01	0.50
**Breakfast cereals**	2.08	0.00	123.77	0.59
**Butter, margarine, and spreads**	13.73	0.00	132.64	1.62
**Canned foods**[a]	0.00	0.00	137.73	0.18
**Canned vegetables, pickled cucumber and beetroot**[a]	6.68	0.00	144.12	0.97
**Cheese**	65.18	0.00	394.48	6.96
**Cigarettes and cigars**	0.00	0.00	894.53	4.17
**Cocoa**	0.00	0.00	68.64	0.12
**Coffee**	12.86	0.00	408.41	1.77
**Cookies, rusks and bagels**	7.13	0.00	172.87	1.04
**Desserts**	10.59	0.00	339.20	1.86
**Dried bread**	3.16	0.00	178.16	0.59
**Eggs**	8.04	0.00	142.75	1.04
**Fish products, frozen, and canned fish**	9.73	0.00	244.98	1.38
**Fresh-baked bread**	34.18	0.00	462.62	4.00
**Fresh, smoked, and salted fish**	15.34	0.00	451.37	2.50
**Frozen fruits and berries**	0.38	0.00	304.17	0.53
**Frozen potato products**	1.88	0.00	98.92	0.35
**Frozen vegetables**	2.92	0.00	163.53	0.53
**Fruit**	43.06	0.00	482.96	5.09
**Ice cream**	12.83	0.00	373.35	1.87
**Juices, nectars, and juice drinks**	10.99	0.00	306.43	1.74
**Meat products**	27.63	0.00	371.93	3.16
**Milk and cream**	43.43	0.00	420.87	4.94
**Mushroom**	0.00	0.00	62.30	0.14
**Mutton, horse and game**	0.00	0.00	90.16	0.08
**Nuts, dried fruits and berries**	8.40	0.00	437.96	1.49
**Other sour milk products**	13.52	0.00	255.86	1.98
**Pastries and confectionery**	31.60	0.00	433.60	3.94
**Pig and bovine meat** [b]	33.52	0.00	267.88	3.96
**Potato**	4.67	0.00	77.54	0.61
**Poultry**	24.23	0.00	379.96	3.00
**Ready-to-eat foods** [c]	53.24	0.00	660.37	6.90
**Ready-to-eat salads**	0.00	0.00	115.12	0.03
**Rice and pasta**	5.25	0.00	162.93	0.68
**Rolled cereals and groats**	2.01	0.00	306.35	0.41
**Root vegetables**	5.55	0.00	173.67	0.76
**Rye flour**	0.00	0.00	43.00	0.01
**Sausages**	20.44	0.00	306.12	2.58
**Seeds and legumes**	0.33	0.00	130.18	0.22
**Shellfish and molluscs**	0.00	0.00	63.32	0.04
**Snacks**	4.44	0.00	182.79	0.84
**Soft drinks and waters**	16.34	0.00	470.83	2.80
**Spices**	3.67	0.00	68.88	0.46
**Sugars and honey**	2.36	0.00	106.78	0.43
**Sweets and chocolate**	32.89	0.00	344.60	4.12
**Tea and herbal drinks**	0.91	0.00	109.28	0.29
**Texmex products**	2.17	0.00	83.47	0.50
**Vegetable oils**	2.59	0.00	89.45	0.44
**Vegetables**	58.55	0.00	416.56	6.42
**Wheat flour**	0.56	0.00	19.66	0.11
**Wine and cider <4.7%**	2.99	0.00	937.02	1.55
**Yogurt**	17.84	0.00	395.50	2.55

[a] *Canned foods include main dishes (e.g., meatballs, chicken soup, ratatouille, tomato soup), and pates (e.g., ham spread, salmon spread). Canned vegetables include products like pickled cucumbers, olives, and preserved tomatoes, among others.*
[b]*Pig and bovine meat include beef and pork as whole meat products and minced meat.*

[c]*Ready-to-eat food includes packaged and service counter sold ready meal portions (such as pasta Bolognese, salmon soup, and ham casserole), frozen meals (e.g., pizzas), and bakery products (e.g., meat pies, paninis)*

Correlation Structures of Products and Time

Our first step in the tensorial approach was to compute the modal covariance matrices for both modes; product groups (55 x 55 matrix) and weeks (52 x 52 matrix). Based on these, we obtained the modal correlation matrices

$$\mathbf{R_1} = \mathrm{diag}(\mathbf{S_1})^{-1/2}\mathbf{S_1}\mathrm{diag}(\mathbf{S_1})^{-1/2}, \qquad \mathbf{R_2} = \mathrm{diag}(\mathbf{S_2})^{-1/2}\mathbf{S_2}\mathrm{diag}(\mathbf{S_2})^{-1/2},$$

where the diag function takes only the diagonal part of its input matrix. The elements of $\mathbf{R_1}$ and $\mathbf{R_2}$ can be interpreted as correlations between the product groups and correlations between the weeks, respectively. The two matrices are visualized in Supplementary Figure 1. Panel A shows that higher (lower) expenditure purchase behaviour tended to be fairly stable, as indicated by positive correlation across all weeks. Especially weeks next to each other correlated heavily, which is a sign of serial correlation (Supplementary Figure 1A). At the same time, the holiday weeks 12, 25, and 51 stand out, due to the understandably different purchase behaviour; these may include different products and amounts compared with everyday life. Similarly, the correlation analysis of expenditure on product groups showed a clear correlation between the expenditures of different product groups such as fruits, vegetables, cheese, milk and cream, sweets, chocolate, ready-to-eat foods, snacks, and soft drinks. Exceptions here are cigarettes, beer, and wine and cider, which can clearly be identified with no correlation or even a negative correlation with other product groups. Instead, they only correlated with each other and also slightly with snacks and soft drinks (Supplementary Figure 1B).

Detecting Atypicalities

Tensorial PCA allows conducting outlier detection simultaneously based on several dimensions [23, 24, 30]. Here, we used it for identifying atypicalities within the data for both time and product dimensions.

From the loadings on weeks $\mathbf{V} \in \mathbf{R}^{52 \times t_0}$, we revealed patterns indicating changes along the season of the year and the weeks next to each other tended to have similar loadings (Supplementary Figure 3A). As often observed also in standard PCA, the first component (i.e. the first column of the loading matrix) represents the “average” over the year, i.e. the loadings for all weeks are roughly equal [29]. The second and the eighth components seemed to have a single week highly loaded, these being Christmas

and Easter, respectively, and these could be interpreted as outlying times. Midsummer was highly loaded in components five and seven, but not alone. This might indicate similar purchase behaviour in some respects as in Christmas ("shared purchasing behaviour"; component 5) and opposite to Christmas ("differential purchasing behavior"; component 7). The nature of these similarities or differences in purchasing behaviour cannot be explored using the time dimension alone. The purchases during holiday seasons were clearly distinguished from the remaining weeks; the only weeks having an absolute loading higher than 0.5 are the weeks of Christmas (week 51), Easter (week 12) and Midsummer (week 25). Not surprisingly, tensorial PCA revealed that the purchase behavior within the holiday seasons is clearly different from other periods of the year, and these periods dominated the loadings of several components.

Simultaneously with estimating the time loadings, tensorial PCA also estimates the loadings $\mathbf{U} \in \mathbf{R}^{55\times p_0}$ for product groups, illustrated in Supplementary Figure 3B. The highest loadings revealed the product groups whose purchases differ most among customers and over time. Many of the products have very low loadings. As the first principal components contain the majority of the variation, we focused on them. Among these, beer and cigarettes clearly stood out with the highest loadings for the first two components. In the first component, beer and cigarettes loaded similarly, which means similarity in their purchase pattern. The second component also loaded highly on beer and cigarettes, but now in the opposite directions. This indicates the presence of purchasing patterns where one of them is bought (e.g. beer) with the other one absent (e.g. cigarettes). Additionally, wine and cider have the single highest loading in component nine. While all previous findings provide interesting insights into purchasing behaviours, these particular products (beer, cigarettes, wine, and cider) provide limited understanding of food purchase patterns as a whole, and thus, we regard them as outlying products for our purposes.

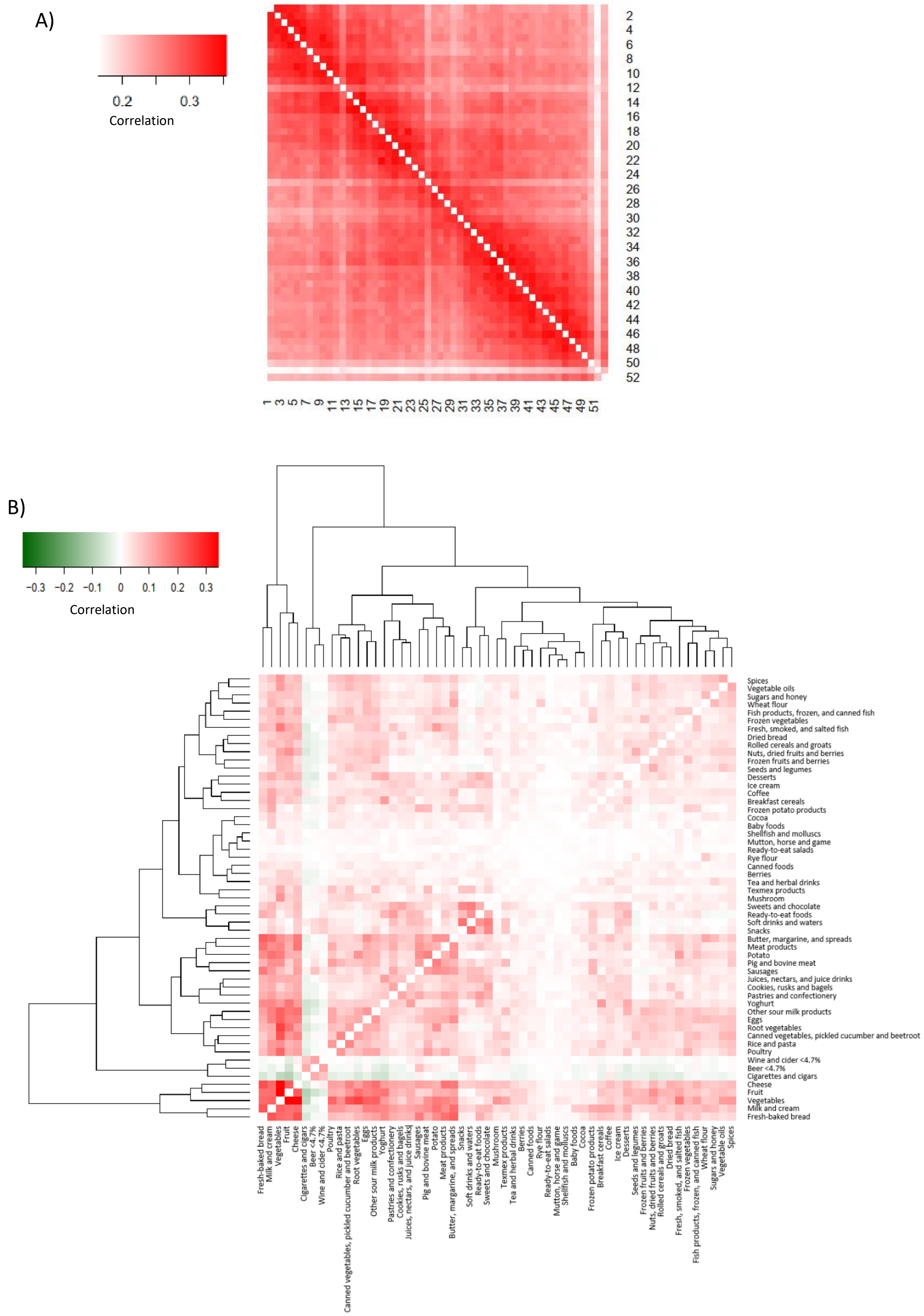

A)
0.2
0.3
Correlation
2 4 6 8 10 12 14 16 18 20 22 24 26 28 30 32 34 36 38 40 42 44 46 48 50 52
1 3 5 7 9 11 13 15 17 19 21 23 25 27 29 31 33 35 37 39 41 43 45 47 49 51
B)
−0.3 −0.2 −0.1 0 0.1 0.2 0.3
Correlation
Spices
Vegetable oils
Sugars and honey
Wheat flour
Fish products, frozen, and canned fish
Frozen vegetables
Fresh, smoked, and salted fish
Dried bread
Rolled cereals and groats
Nuts, dried fruits and berries
Frozen fruits and berries
Seeds and legumes
Desserts
Ice cream
Coffee
Breakfast cereals
Frozen potato products
Cocoa
Baby foods
Shellfish and molluscs
Mutton, horse and game
Ready-to-eat salads
Rye flour
Canned foods
Berries
Tea and herbal drinks
Texmex products
Mushroom
Sweets and chocolate
Ready-to-eat foods
Soft drinks and waters
Snacks
Butter, margarine, and spreads
Meat products
Potato
Pig and bovine meat
Sausages
Juices, nectars, and juice drinks
Cookies, rusks and bagels
Pastries and confectionery
Yoghurt
Other sour milk products
Eggs
Root vegetables
Canned vegetables, pickled cucumber and beetroot
Rice and pasta
Poultry
Wine and cider <4.7%
Beer <4.7%
Cigarettes and cigars
Cheese
Fruit
Vegetables
Milk and cream
Fresh-baked bread

Figure S2. Correlation matrices of shopping data on weeks (A) and expenditures (B). In A, the correlations are arranged by time, and overall there is a clear correlation in the shopping pattern throughout the weeks, and this correlation is even higher between weeks next to each other. Also, the weeks 12, 25, and 51 are outliers with smaller correlations than the others. In B, the product groups are arranged by similarity by means of hierarchical cluster analysis on, i.e. strongly correlating product groups are adjacent to each other. Beer, cigarettes, and wine and cider stand out in this analysis as they correlate with each other but not with other products. Color keys for the correlations given for both subfigures.

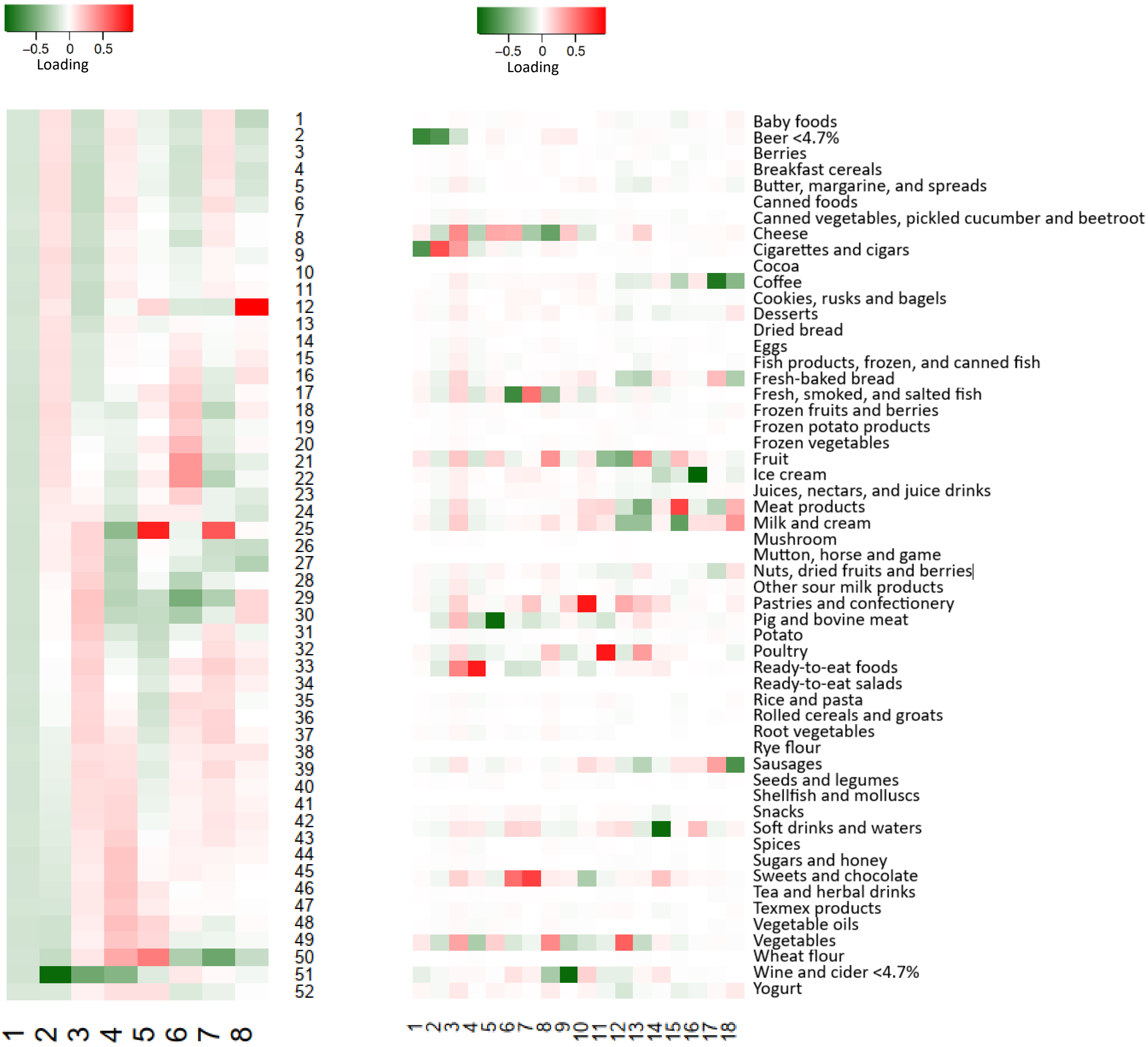

Figure S3. Several first columns of the loading matrices for weeks (A) and for product groups (B) illustrated using heatmaps. In Figure A, the weeks 51, 25, and 12 clearly stand out. In Figure B, most of the product loadings are very small, but some are clearly specific to a certain principal component. For example, cigarettes and beer have the highest absolute loadings for the first two PC, and wine and cider in the ninth PC. Color keys for the loadings given above both subfigures.

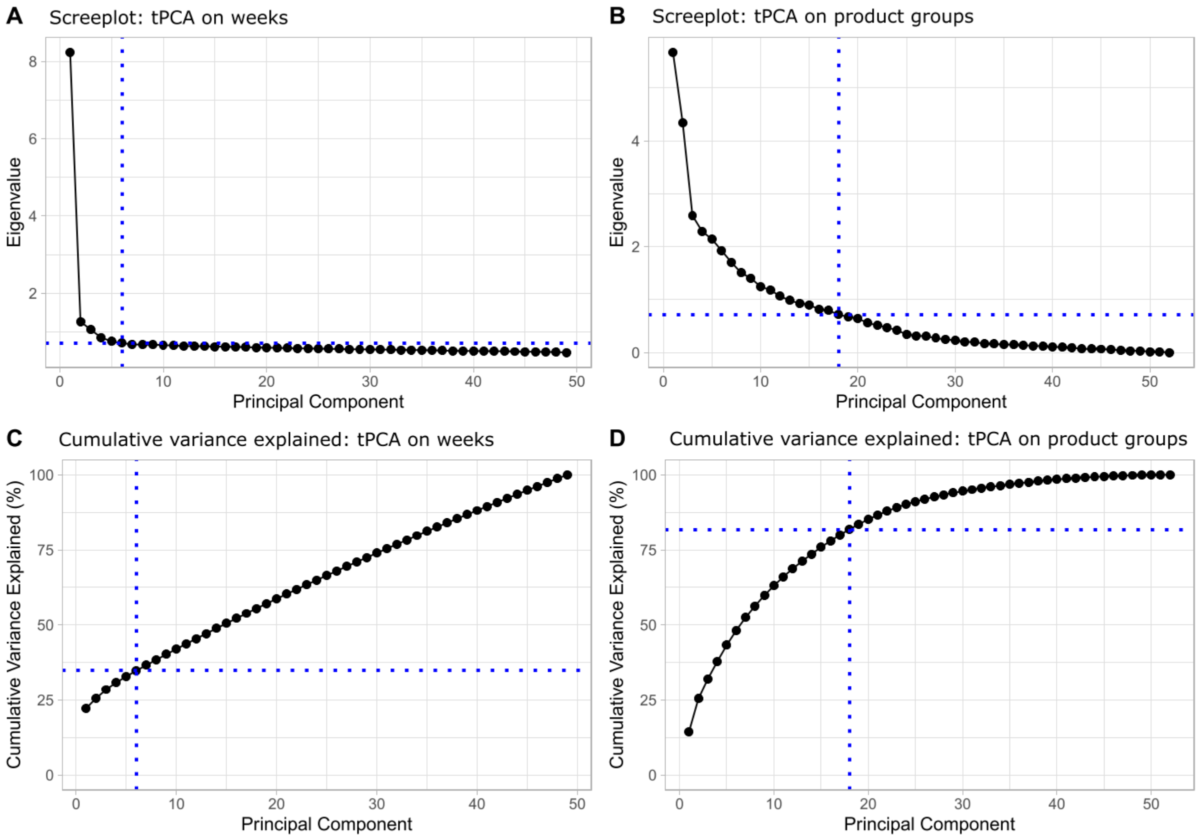

Figure S4: Scree plots and the cumulative variance explained illustrated for the tensorial PCA results. The scree plots show the eigenvalues of the tensorial PCA analysis on A) week -dimension and B) in product groups -dimension, while the cumulative variance explained based on the tensorial PCA analysis are illustrated in C) for the week dimension and D) for the product groups. Dashed lines show the cutoff for the number of significant components, identified with the augmentation method.

Figure S5. Next three pages give eighteen subfigures illustrating the various purchasing behaviour of individuals. Each figure shows the average money spent on each product and each week for the groups of people whose shopping behaviour, i.e. PCs for the specified week and product pair, is within 10% highest (left) or 10% lowest (right). Only the products with absolute loading higher than 0.3 at least for one of the product PC components are illustrated. Color keys for the loadings given above each subfigure.

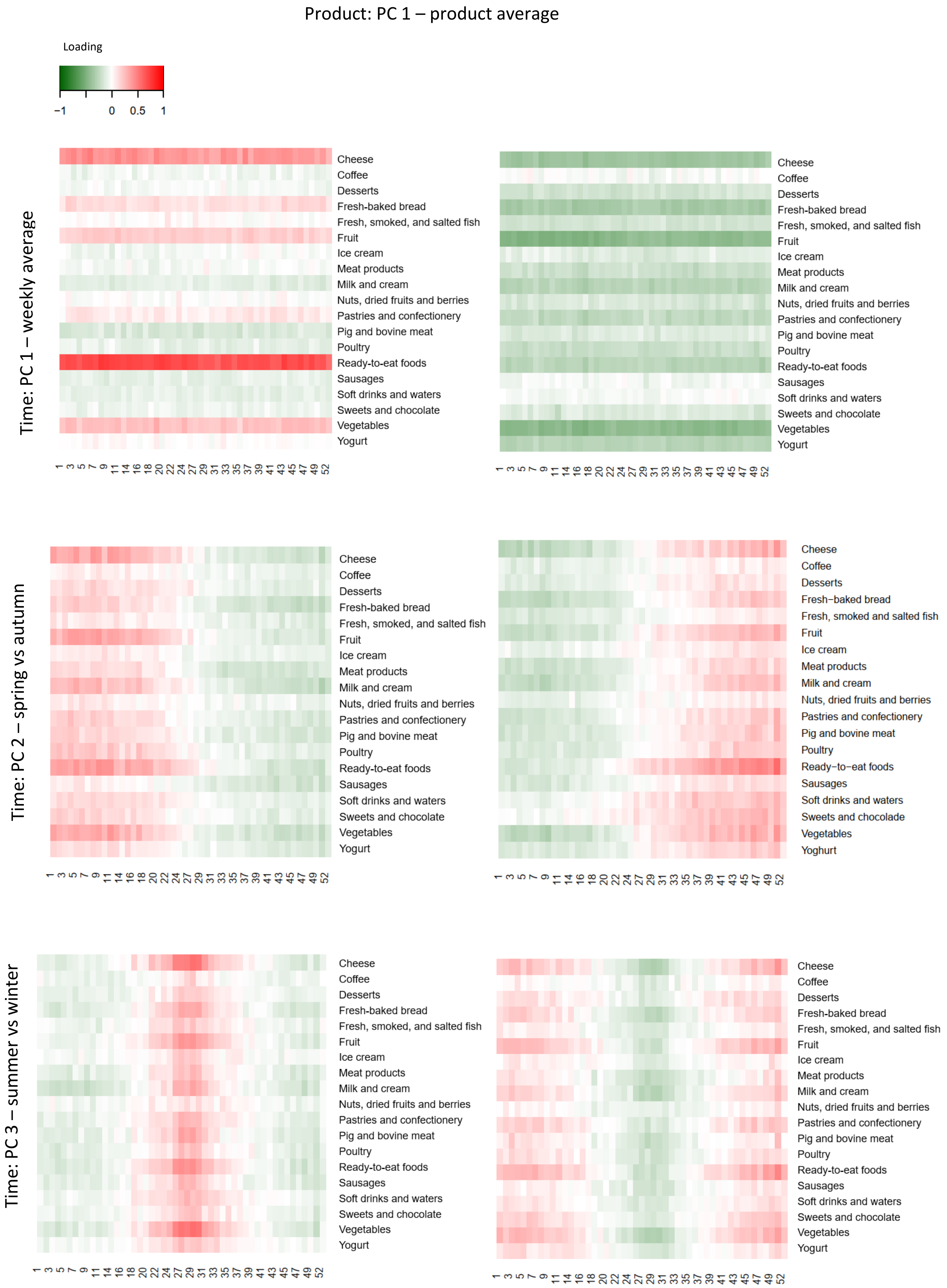
Product: PC 1 – product average
Loading
−1 0 0.5 1
Time: PC 1 – weekly average
Time: PC 2 – spring vs autumn
Time: PC 3 – summer vs winter
Cheese
Coffee
Desserts
Fresh-baked bread
Fresh, smoked, and salted fish
Fruit
Ice cream
Meat products
Milk and cream
Nuts, dried fruits and berries
Pastries and confectionery
Pig and bovine meat
Poultry
Ready-to-eat foods
Sausages
Soft drinks and waters
Sweets and chocolate
Vegetables
Yogurt
Fresh−baked bread
Fresh, smoked and salted fish
Ready−to−eat foods
Sweets and chocolade
Yoghurt
1 3 5 7 9 11 14 16 18 20 22 24 27 29 31 33 35 37 39 41 43 45 47 49 52

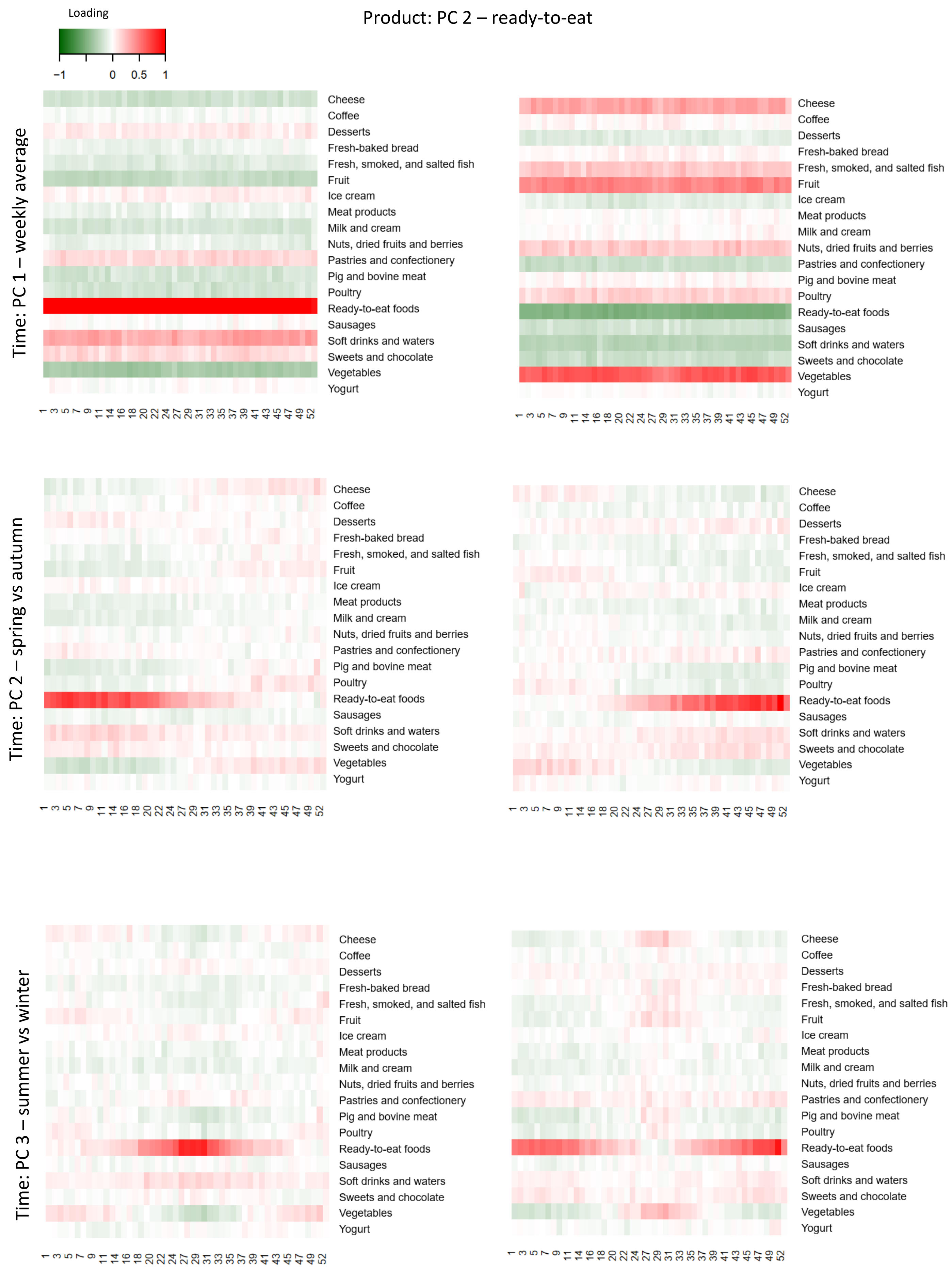

Product: PC 2 – ready-to-eat
Loading
−1
0
0.5
1
Time: PC 1 – weekly average
Time: PC 2 – spring vs autumn
Time: PC 3 – summer vs winter
Cheese
Coffee
Desserts
Fresh-baked bread
Fresh, smoked, and salted fish
Fruit
Ice cream
Meat products
Milk and cream
Nuts, dried fruits and berries
Pastries and confectionery
Pig and bovine meat
Poultry
Ready-to-eat foods
Sausages
Soft drinks and waters
Sweets and chocolate
Vegetables
Yogurt
1 3 5 7 9 11 14 16 18 20 22 24 27 29 31 33 35 37 39 41 43 45 47 49 52

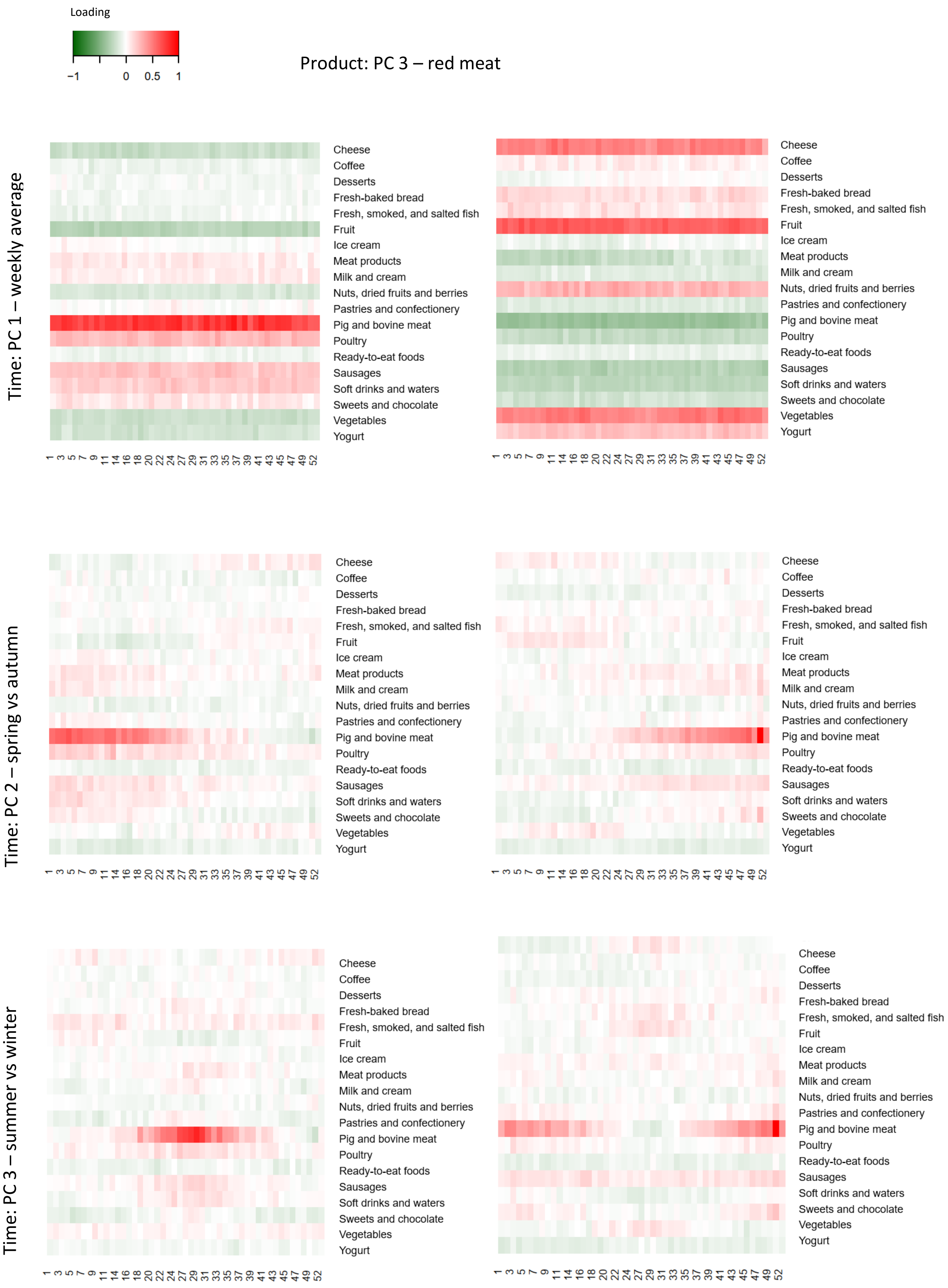

Loading
−1
0
0.5
1
Product: PC 3 – red meat
Time: PC 1 – weekly average
Time: PC 2 – spring vs autumn
Time: PC 3 – summer vs winter
Cheese
Coffee
Desserts
Fresh-baked bread
Fresh, smoked, and salted fish
Fruit
Ice cream
Meat products
Milk and cream
Nuts, dried fruits and berries
Pastries and confectionery
Pig and bovine meat
Poultry
Ready-to-eat foods
Sausages
Soft drinks and waters
Sweets and chocolate
Vegetables
Yogurt
1 3 5 7 9 11 14 16 18 20 22 24 27 29 31 33 35 37 39 41 43 45 47 49 52

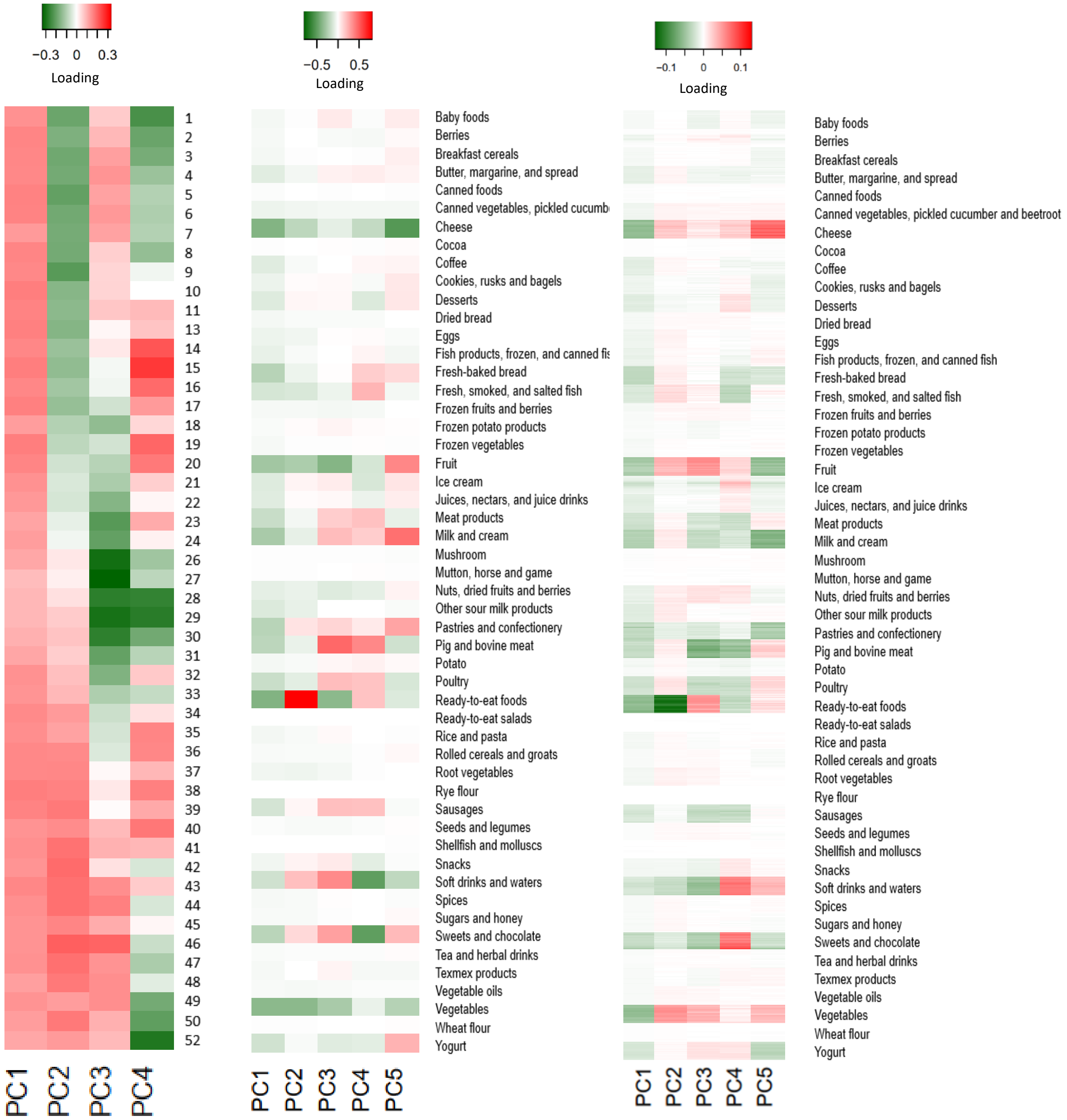

Figure S6. Illustration of loadings computed with traditional PCA. A) On the left, data are arranged as weekly data, in which the spent money has been summed over all products. The loadings of weeks next to each other are typically similar. While the first component represents average purchases throughout the year, the second shows the increased/decreased amount of purchases over time, and the loadings of the third component show the pattern when purchasing behaviour is different in summer than in winter. B) Data are product-wise, i.e. the spent money has been summed over all weeks. Again, the first component illustrates approximately the average pattern, while the second especially highlights

the ready-to-eat food, and the third pig and bovine meat. C) All products and weeks have been combined, and the resulting figure includes loadings separately for each product-week pair (for clarity, the names are only printed once, but the colourmap includes all loadings of weeks 1-52 on top of each other within each product). The dominant feature is formed by the products that have different shopping patterns between individuals and the heatmap is very similar to that in Figure B. Color keys for the loadings given above each subfigure.

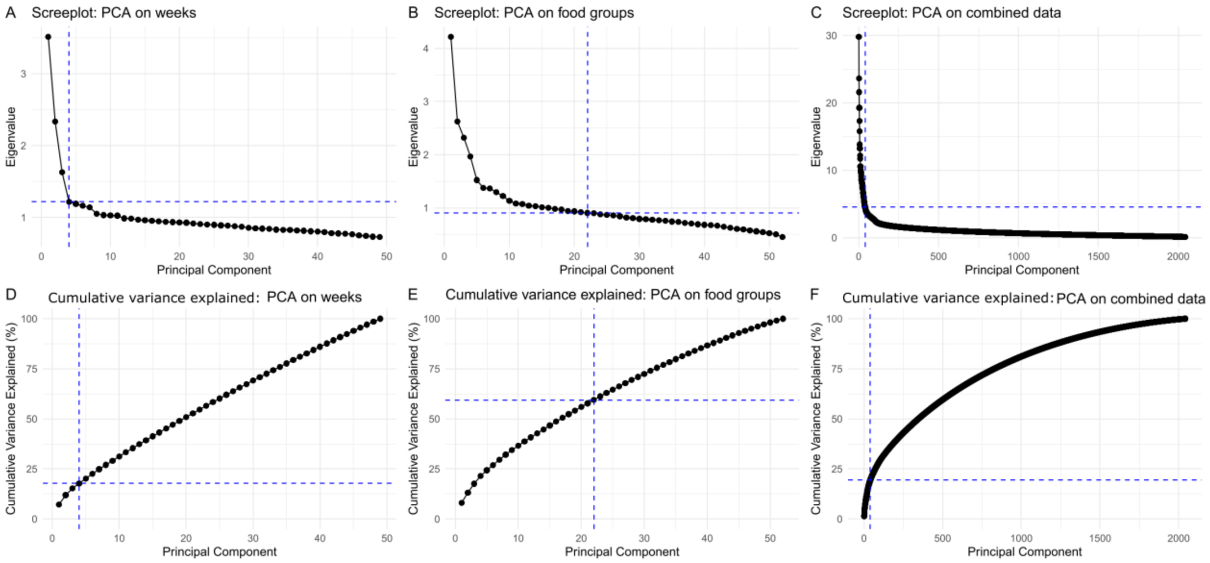

Figure S7. Illustration of scree plots and the cumulative variance explained plots of the standard PCA results. On the left, data is arranged as weekly data, in which the spent money has been summed over all products, and Figure A is the scree plot of such data, while Figure D illustrates the cumulative variance explained. Similarly, in the middle, Figures B and E illustrate the results of product-wise data, and Figures C and F illustrate the results of the combined data. Dashed lines in each figure show the number of principal components that were detected as significant using the augmentation method.

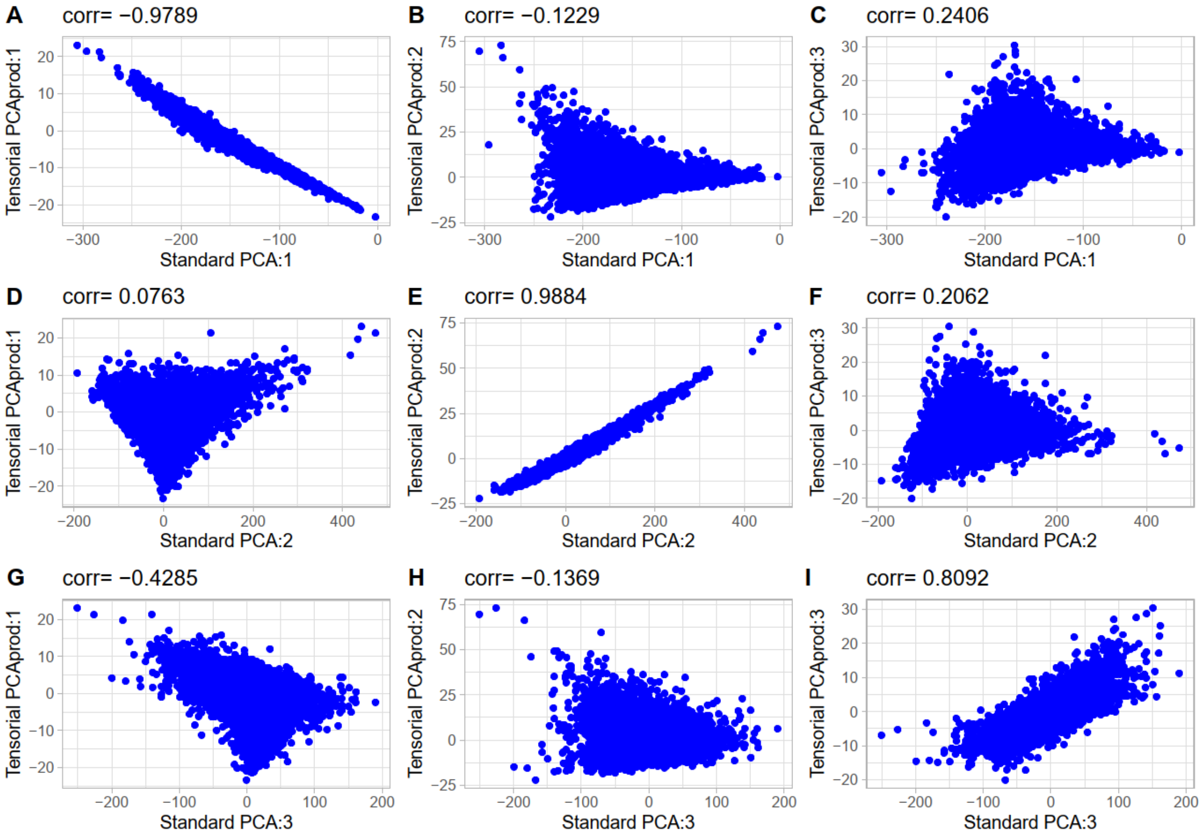

Figure S8. Comparison between the principal component scores of individuals. The focus on tensorial analysis is the first time component, while the product component varies between 1 and 3 within figures (each product component corresponds to a single column of subplots). We aimed to determine whether the first three PCA scores of the products summed across years revealed the same information as product PCs 1-3 of the tensorial analysis. The diagonal plot (A) illustrates a clear correlation between the standard PC(PC_{prod}=1) and tensorial PC (PC1 - product average, PC1 - weekly average) as well as (E) standard PC(PC_{prod} =2) and tensorial PC(PC2 - ready-to-eat, PC1 - weekly average).
